# Lead induces cell-autonomous proliferation and metabolic reprogramming of hepatocytes

**DOI:** 10.1038/s41419-025-08134-6

**Published:** 2025-11-10

**Authors:** Marina Serra, Alfredo Smiriglia, Cristina Migliore, Andrea Caddeo, Nicla Lorito, Gabriele Tani, Giorgia Zedda, Amedeo Columbano, Andrea Perra, Silvia Giordano, Marta Anna Kowalik, Andrea Morandi

**Affiliations:** 1 Department of Biomedical Sciences, University of Cagliari, Monserrato, Italy; 2 Department of Experimental and Clinical Biomedical Sciences, University of Florence, Florence, Italy; 3 Department of Oncology, University of Torino, Candiolo, Turin Italy; 4 Candiolo Cancer Institute, FPO-IRCCS, Candiolo, Turin Italy

**Keywords:** Cell biology, Cell division

## Abstract

Reprogramming of energy metabolism is widely recognized as a hallmark of cancer cells. However, recent evidence indicates that metabolic reprogramming also occurs in vivo in differentiated rat hepatocytes following administration of the primary mitogen lead nitrate (LN). It remains unclear whether this phenomenon results from a direct action of LN on hepatocytes or is mediated by non-parenchymal liver cells. In our study, we investigated the cell-autonomous effects of LN using immortalized non-tumorigenic rat (RNT) and human (THLE-2) hepatocytes. LN treatment induced cancer-like metabolic features in non-tumorigenic hepatocytes, including increased glycolysis, activation of both oxidative and non-oxidative pentose phosphate pathways (PPP), and reduced oxidative phosphorylation (OXPHOS). Additionally, LN increased several targets of the transcription factor nuclear factor (erythroid-derived 2)-like 2 (NRF2), a key regulator of cellular defense against stress. We found that activation of the Kelch-like ECH-associated protein 1 (KEAP1)-NRF2 pathway was associated with increased hepatocyte proliferation. Importantly, silencing NRF2 completely abolished the LN-induced metabolic reprogramming. In contrast, triiodothyronine (T3), a liver mitogen that does not activate NRF2, failed to trigger metabolic reprogramming. Overall, our findings demonstrate that LN directly drives both proliferation and metabolic reprogramming in hepatocytes, independently of microenvironmental or immune signals. NRF2 plays a central role as a key driver of these cancer-like metabolic shifts, even in non-tumorigenic cells.

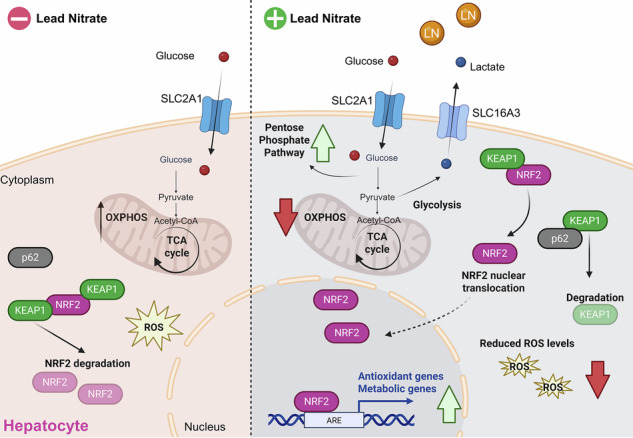

## Introduction

The development of cancer is a multistep process whereby neoplastic cells acquire several functional traits, collectively known as “hallmarks of cancer”, which enable them to proliferate, survive, and disseminate [1]. Among them, a key feature of cancer cells is metabolic reprogramming. In contrast to normal cells, neoplastic cells exhibit a distinctive form of metabolism and undergo a significant metabolic shift, known as aerobic glycolysis or “Warburg effect”, whereby glucose utilization is prioritized, and oxidative phosphorylation (OXPHOS) is frequently impaired. This provides rapidly dividing tumor cells with metabolic intermediates that serve as biosynthetic precursors [2]. Extensive metabolic rewiring has been documented in many cancers, including hepatocellular carcinoma (HCC) [3]. However, disturbances in energy metabolism can also occur in non-malignancy conditions [4]. A common trait of cells engaging in aerobic glycolysis, whether they are cancerous or not, is rapid proliferation [5]. Therefore, comparing how mitogenic chemicals change metabolism in normal hepatocytes with the rewiring seen in cancerous hepatocytes represents a valuable approach to better understand the critical determinants of HCC onset and progression.

In this context, the Kelch-like ECH-associated protein 1-nuclear factor (erythroid-derived 2)-like 2 (KEAP1-NRF2) system, which represents the main intracellular defense against environmental stress, has been identified as an important regulator of proliferation and metabolic reprogramming in several human tumors [6]. NRF2 activation enhances glucose uptake and directs it towards the pentose phosphate pathway (PPP), by regulating the expression of the key enzymes involved in the oxidative and non-oxidative branches of the PPP [7]. A considerable body of evidence has indicated that the activation of NRF2 through various mechanisms is a common occurrence in primary tumors and is associated with the promotion of cancer progression [8]. Importantly, NRF2 induces the expression of glucose-6-phosphate dehydrogenase (G6PD), the rate-limiting enzyme of the PPP, required to support anabolic demands and to orchestrate metabolic deregulation [9]. In agreement, metabolic changes leading to increased G6PD expression are associated with a higher proliferative capacity of preneoplastic or neoplastic lesions [10].

Although this metabolic reprogramming has been observed in many cancer cells, including transformed hepatocytes, no in-depth studies have been undertaken to assess whether normal hepatocytes undergo metabolic reprogramming, similar to that observed in cancer cells, when stimulated to proliferate. This question is relevant since it has been recently suggested that metabolic reprogramming is not a unique property of neoplastic cells [11], demonstrating that NRF2-dependent metabolic reprogramming can also occur in differentiated proliferating cells after treatment with the hepatic mitogen lead nitrate (LN) [12], a heavy metal contaminant, whose exposure represents a major risk to human health. LN is an established direct mitogen in rodent models, inducing synchronized hepatocyte proliferation without causing necrosis or liver injury [12–14] and a powerful tool to study early regenerative and preneoplastic events because it triggers both proliferation and oxidative stress. Interestingly, NRF2 activation did not occur in other protocols of hepatocyte proliferation, such as treatment with triiodothyronine (T3) or partial hepatectomy (PH) [11]. To our knowledge, no compounds other than LN can reproduce in the healthy liver the complete and faithful complex of biochemical-metabolic alterations generally observed in preneoplastic lesions or advanced HCC.

Since the acute LN model offers a rapid and useful in vivo model to identify and compare the metabolic dependencies of preneoplastic hepatic lesions, HCC and those of normal proliferating hepatocytes [11], we explored the mechanisms leading to metabolic reprogramming in normal hepatocytes. Two key questions were addressed in the present study: 1) is hepatocyte proliferation a direct effect of LN on hepatocytes or is it mediated by non-parenchymal cells, as suggested by previous studies [14–16]?; 2) is KEAP1-NRF2 pathway activation and metabolic reprogramming a consequence of microenvironment/hepatocyte crosstalk, as suggested by several studies [17, 18], or can also occur as a direct effect exerted on hepatocytes?

To this aim, we took advantage of 1) RNT, a rat immortalized non-tumorigenic hepatocyte cell line obtained by perfusion of rat livers exposed to the Resistant-Hepatocyte protocol of hepatocarcinogenesis [19, 20], and 2) THLE-2, a human immortalized non-tumorigenic cell line displaying features of typical mature hepatic epithelial cells. Crucially, in this study, we exploit LN to explore the mechanisms intrinsic to hepatocytes and, for the first time, we demonstrate that LN triggers NRF2‑driven metabolic reprogramming even without interactions with non‑parenchymal and immune cells.

## Methods

### Cell cultures

RNT cells were isolated from rats treated with 2-acetylaminofluorene (2-AAF) and subjected to partial hepatectomy (PH), which did not develop tumors, as described in Angioni et al. [20]. RNT cells were maintained in RPMI medium (Thermo Fisher Scientific, 31870-025, Lot n° 3023277) supplemented with 10% fetal bovine serum (FBS) (Euroclone, ECS5000L, Lot n° EUS00NS), 2 mM glutamax (Thermo Fisher Scientific, 35050061, Lot n° 3224711), and 1% penicillin/streptomycin (Sigma Aldrich, P081, Lot n° 0000205667). THLE-2 cells (ATCC, CRL-2706, RRID: CVCL_3803) were grown in BEGM Bronchial Epithelial Cell Growth Medium BulletKit (Lonza/Clonetics Corporation, CC-3170, Lot n° 0001289756) supplemented with 5 ng/mL epidermal growth factor (EGF) (Sigma Aldrich, E9644, Lot n° 0000374424), 70 ng/mL Phosphoethanolamine (Sigma Aldrich, P0503, Lot n° BCCH0267), and 10% FBS (Euroclone, ECS1800D, Lot n° EUS42061623). Culture plates were precoated with a mixture of 0.01 mg/mL fibronectin (Corning, 354008, Lot n° MQC3024021), 0.03 mg/mL rat tail collagen type I (Corning, 354236, Lot n° 2160002), and 0.01 mg/mL bovine serum albumin (BSA) (Sigma Aldrich, A7979, Lot n° 0000363804) dissolved in culture media.

### RNA extraction and quantitative reverse transcription PCR (qRT-PCR) analysis

Total RNA was extracted from cells treated with lead nitrate (LN, 100 or 500 µM) (Sigma Aldrich, 228621, Lot n° BCCK6767) or triiodothyronine (T3, 100 nM) (Sigma Aldrich, T2752, Lot n° SLBK1093V) and quantified with NanoDrop (Thermo Fisher Scientific). 2 μg of RNA were reverse-transcribed using High-Capacity cDNA Reverse Transcription Kit (Thermo Fisher Scientific, 4368814). The mRNA expression levels were assessed by qRT-PCR analysis using 10 ng of cDNA mixed with 2X TaqMan Gene expression Master Mix (Thermo Fisher Scientific, 4369016) and 20X specific TaqMan gene expression assays (Thermo Fisher Scientific) with an ABI PRISM 7300 Thermocycler. Reactions were run for 40 cycles. Samples were called “detected” when the target showed a Ct < 35 in at least two technical replicates. Signals with Ct ≥ 35 or no amplification were classified as ‘undetermined’ and are not interpreted quantitatively. qRT-PCR primers used were listed in Supplemental Table S1. *β-actin* was used as an endogenous control. Relative quantification for each gene was calculated by the 2^-ΔΔCt^ method.

### Small interfering RNA (siRNA)-targeted gene silencing

A pool of 30 distinct siRNAs specific for *NRF2* (siNRF2) was purchased by siTOOLs BIOTECH (si-G050-83619 for RNT cells and si-G050-2551 for THLE-2 ones). Briefly, RNT or THLE-2 cells were cultured on 6-well plates and grown to 70% confluence before being transfected. After 24 hours of cell culture at 37 °C, cells were transfected with 25 nM siNRF2 or the negative control (non-targeting small interfering RNA, siCTR, scramble) using Lipofectamine RNAiMAX Reagent (Thermo Fisher Scientific, 13778-150) according to the manufacturer’s protocol. After 48 hours of treatment, the medium containing the transfection complex was replaced with a medium containing LN or normal medium for an additional 24 hours. NRF2-knockdown cells were established for the indicated experiments after 72 hours from the cell transfection.

### ^3^[H]-thymidine incorporation assay/Proliferation assay

To evaluate [^3^H]-thymidine incorporation, hepatocytes cells (5×10^4^ cells/well) were seeded into 12-well plates and serum-starved for 24 hours. Subsequently, the cells were treated with 100 µM LN or 100 nM T3 in culture medium for 24 hours in RNT cells and 48 hours in THLE-2 cells. [^3^H]-thymidine (Perkin Elmer, NET027001MC, 0.5 µCi/well, Lot n° 202501) was added for the final 6 hours for RNT and 24 hours for THLE-2 cells of incubation at 37°C. Cells were washed twice in PBS before the addition of 300 µL of 10% trichloroacetic acid (TCA) (Sigma-Aldrich, T4885) for 15 minutes at room temperature and then washed twice with 300 µL of 10% TCA. Cells were subsequently lysed with 300 µL of NaOH (0.1 M) (Sigma-Aldrich, 1.60309) and transferred to scintillation vials. The radioactive signal was counted on the scintillation counter and normalized on protein content.

### Pentose phosphate pathway (PPP) assay

To evaluate PPP flux, RNT cells (5×10^4^ cells/well) were seeded into 12-well plates. The day after, cells were treated with 100 µM of LN for 24 hours. Subsequently, PPP activity was evaluated by using radioactive glucose labeled in position 1 [1-^14^C] (Perkin Elmer, NEC043X050UC, Lot n° 2734965) or in position 6 [6-^14^C] (Perkin Elmer, NEC045X050UC, Lot n° 2140007). ^14^CO_2_ developed from [1-^14^C]-glucose oxidation originates from the PPP or the tricarboxylic acid cycle, whereas ^14^CO_2_ released from [6-^14^C]-glucose originates only from tricarboxylic acid cycle. 2 μCi [1-^14^C]-glucose or 2 μCi [6-^14^C]- glucose were added for 1 hour to the cells (two different plates of same sample treated in parallel). PPP CO_2_ production is therefore revealed by subtracting the radioactive signal derived from [1-^14^C]-glucose to that of [6-^14^C]-glucose. Such values were then normalized on total cell protein content and shown as fold change. Each dish had a taped piece of Whatman paper facing the inside part of the dish wetted with 200 μL of phenylethylamine-methanol (1:1) to trap the CO_2_. Then 150 μL of 4 M H_2_SO_4_ were added to cells. Finally, Whatman paper was removed and transferred to scintillation vials for counting. Radioactive signal was measured by liquid scintillation counting and normalized for protein content.

### Reactive Oxygen Species (ROS) analysis

RNT cells (5×10^4^ cells/well) were seeded into 12-well plates under the conditions described in Figure Legends. Cells were stained with 5 μM of 5,6-Carboxy-2’,7’-Dichlorofluorescein Diacetate probe (DCFDA) (Sigma-Aldrich, D6883) or 5 μM of CellROX probes (Thermo Fisher Scientific, C10444, Lot n° 2871991) and incubated at 37°C in the dark for 30 minutes. Then, cells were lysed with RIPA buffer (Thermo Fisher Scientific, 89900, Lot n° ZA382954) and fluorescence was measured on a microplate reader at 485/535 nm Excitation (Ex)/Emission (Em). Fluorescence was normalized on protein content.

### Flow cytometry analysis (FACS)

RNT cells (5×10^4^ cells/well) were seeded into 12-well plates and treated as described in Figure Legends. The day after, cells were stained at 37°C for 30 minutes with 5 μM of DCFDA and 5 μM of CellROX probes (Thermo Fisher Scientific, C10444, Lot n° 2871991) for ROS content, and for 1 hours with 5 μM BODIPY^581/591^-C_11_ (Thermo Fisher Scientific, D3861) to evaluate lipid peroxidation. Live cells resuspended in PBS with 0.1% FBS were subjected to FACS analysis using FACSCanto II (BD Biosciences). 1×10^4^ cells were analyzed for the median fluorescence intensity of specific probes.

### GSH/GSSG measurement

RNT cells were seeded in 96-well plates (1×10^4^ cells/well) and subjected to the LN-treatment (100 μM) for 24 hours treatment with LN. GSH and GSSG levels were measured according to manufacturer’s instructions (GSH/GSSG-Glo Assay, Promega V6611, Lot n° 0000503970). Luminescence was read using a luminometer and was proportional to the amount of GSH. A twofold adjustment was required for GSSG concentration because each mole of oxidized GSSG upon reduction produces two moles of GSH in this assay. The GSH/GSSG ratio was calculated from luminescence measurements (in relative light units, RLU) normalized on protein content.

### Immunofluorescence staining

RNT cells (5×10^3^ cells/well) were seeded into Nunc™ Lab-Tek™ Chamber Slides and treated with LN (100 µM) and ivermectin (IVE, 5 µM) (Sigma-Aldrich, I8858, Lot n° MKCV0451) for 24 hours to evaluate NRF2 nuclear translocation. The day after, cells were fixed in ice-cold ethanol for 10 minutes and permeabilized with 0.2% Triton X-100 (Sigma-Aldrich, T8787, Lot n° 0000302971) in PBS for 10 minutes followed by blocking for 1 hour with 5% BSA in PBS. Subsequently, cells were incubated overnight at 4°C with primary antibodies anti-NRF2 (Proteintech, 16396-1-AP, Lot n° 00129341) diluted 1:200. The day after, cells were washed three times with PBS and incubated with a 1:2000 dilution of anti-Rabbit IgG Alexa Fluor 488 (Thermo Fisher Scientific, A11034, Lot n° 2861864) in 5% BSA-containing PBS for 1.5 hours at room temperature in the dark. For nuclei staining, fixed cells were incubated with DAPI (Thermo Fisher Scientific, D3571) for 10 minutes at room temperature. Sample images were acquired using a TCS SP8 microscope (Leica Microsystems) with LAS-AF image acquisition software.

### Confocal image acquisition and analysis

RNT cells were plated at a concentration of 1×10^4^ per well in a Nunc™Lab-Tek™Chamber Slide System coverglass (ThermoFisher Scientific, 155383). 24-hour post-treatment with LN (100 µM), cells were stained at 37 °C for 30 min with MitoTracker Green (ThermoFisher Scientific, M7514, Lot n° 2379392) used to assess mitochondrial mass and damage, and DAPI (Thermo Fisher Scientific, D3571) for nuclei staining and subsequently washed an additional 3 times in PBS. When the incubation with the probes was completed, images were acquired using a TCS SP8 microscope (Leica Microsystems) with LAS-AF image acquisition software.

Quantification of MitoTracker Green fluorescence intensity was performed using Leica-Las-X-suite software. Mitochondria-Analyzer plugin in ImageJ v.1.54k was used to determine the average mitochondrial aspect ratio (major axis length ÷ minor axis length) and form factor, as described in [21]. The aspect ratio reflects mitochondrial elongation, while the form factor also accounts for perimeter measurements, making it more sensitive to curvature and the irregular shapes characteristic of filamentous mitochondria. Higher values of both form factor and aspect ratio indicate increased mitochondrial branching and structural complexity. Three representative cells per field of view were chosen from 3 technical replicates of at least 3 independent biological experiments.

### Nuclear/Cytoplasmic Fractionation

RNT cells were washed twice with PBS and incubated on ice for 10 minutes in a buffer containing 15 mM KCl (Sigma-Aldrich, P9541), 10 mM HEPES pH 7.6 (AppliChem, A3724), 2 mM MgCl₂ (Sigma-Aldrich, M8266), 0.1 mM EDTA (Sigma-Aldrich, E7889), 0.1% NP-40 (Thermo Fisher Scientific, 28320), and 1X phosphatase and protease inhibitor cocktail (Thermo Fisher Scientific, 78444). Following incubation, samples were centrifuged at 10,000 rpm for 10 minutes. The supernatant, containing the cytoplasmic fraction, was collected. The pellet was then incubated on ice for 5 minutes in a buffer composed of 2 mM KCl, 25 mM HEPES pH 7.6, 0.1 mM EDTA, and 1X phosphatase and protease inhibitor cocktail. Subsequently, the pellet was resuspended in a second buffer containing 25 mM HEPES pH 7.6, 0.1 mM EDTA, 20% glycerol (Sigma-Aldrich, G5516), and 1X phosphatase and protease inhibitor cocktail. After a final centrifugation at maximum speed, the supernatant containing the nuclear fraction was collected.

### Western blotting analysis

Cells were washed with PBS and lysed on ice with RIPA buffer (Thermo Fisher Scientific, 89900, Lot n° ZA382954) supplemented with protease and phosphatase inhibitors (Sigma-Aldrich, P8340 and P0044, respectively), and protein concentrations were measured by BCA (Sigma-Aldrich, 1003290033) method. Protein samples were mixed with Laemmli Sample Buffer 4X (Biorad, 1610747), boiled for 5 minutes at 95 °C, and 30–40 μg of cell lysate were loaded in precast SDS-PAGE (sodium dodecyl sulfate–polyacrylamide gel electrophoresis) gels (Biorad, 456-8096) and then transferred onto nitrocellulose membrane by Trans-Blot Turbo Transfer Pack (Biorad, 170-4157). The immunoblots were incubated in non-fat dry milk 5%, tween-20 0.05% in PBS at room temperature for 1 hour, and then probed with primary and appropriate secondary antibodies. Antibodies used for western blotting analysis are indicated in Supplemental Table S2. Full and uncropped blots are provided in Supplementary Material.

### Seahorse XFe96 Metabolic Assays

#### Mito Stress Assay

Hepatocytes were seeded in XFe96 cell culture plates with 1.5–2×10^4^ cells per well (10 technical replicates) and subjected to the XF Mito Stress test (Agilent Technologies, 103010-100, Lot n° 17412536). The day after, cells were treated with LN (100 µM) for 24 hours in their culture medium that was replaced post-treatment with XF RPMI medium supplemented with 11 mM glucose, 2 mM glutamine, and 1 mM sodium pyruvate. Cells were incubated for 1 hour at 37 °C in a non-CO_2_ incubator before the analysis. The oxygen consumption rate (OCR) was quantified using the Seahorse Extracellular Flux Analyzer (XFe96, Agilent Technologies). Together with OCR, values of the extracellular acidification rate (ECAR), which is dependent on the mitochondrial-derived CO_2_ and on that of glycolysis, were also recorded. The addition of the ATP synthase inhibitor oligomycin (Oligo, 1.5 μM), the proton uncoupler FCCP (1 μM), the respiratory complex I inhibitor rotenone (Rot, 0.5 μM), and the respiratory complex III inhibitor antimycin A (AA, 0.5 μM) was carried out at the times indicated. Protein quantification was used to normalize the results. Basal respiration is calculated as the last rate measurement before Oligo injection minus the non-mitochondrial respiration rate. Maximal respiration is calculated as the maximum rate measurement after FCCP injection minus the non-mitochondrial respiration rate. ATP production is calculated as the decrease in OCR upon Oligo injection and represents the portion of basal respiration used to drive ATP synthesis.

#### Glycolysis Stress Test

RNT cells were seeded in XFe96 cell culture plates with 1.5–2×10^4^ cells per well (7 technical replicates) and subjected to the XF Glycolysis Stress test (Agilent Technologies, 103020-100). The day after, cells were treated with LN (100 µM) for 24 hours. Post-treatment, the culture medium was replaced with 180 μL of XF RPMI supplemented with 2 mM glutamine and without glucose or pyruvate. Cells were incubated for 1 hour at 37 °C in a non-CO_2_ incubator to allow them to pre-equilibrate with the XF RPMI. For the glycolytic function assessment, ECAR was quantified using the Seahorse XFe96 Analyzer. The analysis was performed in real-time by measuring ECAR after the sequential injections of a saturating concentration of glucose (10 mM), Oligo (1.5 μM), and 2-Deoxy-D-glucose (2-DG, 50 mM), which inhibits glycolysis by competitively binding the first enzyme in the glycolytic pathway (i.e., hexokinase), confirming that the ECAR produced is due to glycolysis. Protein quantification was used to normalize the results. Basal glycolysis is calculated as the last rate measurement after glucose injection subtracted of the non-glycolytic acidification rate. Glycolytic capacity is calculated as the maximum rate measurement after Oligo injection minus the non-glycolytic acidification rate.

#### Glycolytic Rate Assay

RNT cells were seeded in XFe96 cell culture plates with 1.5–2×10^4^ cells per well (7 technical replicates) and treated with LN (100 μM) for 24 hours. Post-treatment, the cells were subjected to the XF Glycolytic Rate assay (Agilent Technologies, 103344-100, Lot n° 16961340). The culture medium was replaced with 180 μL of XF RPMI supplemented with 10 mM glucose and 2 mM glutamine. Cells were incubated for 1 hour at 37 °C in a non-CO_2_ incubator to allow them to pre-equilibrate with the XF RPMI. The Glycolytic Rate Assay discriminates between ECAR that is dependent on mitochondrial-derived CO_2_ and that of glycolysis by concomitantly measuring the amount of OCR, thereby calculating the total proton efflux rate (PER). This analysis is performed by measuring in real-time ECAR and OCR after Rot/AA (0.5 μM) and 2-DG (50 mM) administration. Inhibition of mitochondrial function by Rot/AA allows the calculation of mitochondrial-associated acidification. The subtraction of mitochondrial acidification (MitoPER) from the total PER results in the Glycolytic Proton Efflux Rate (GlycoPER). Protein quantification was used to normalize the results.

### Oroboros O2k-FluoRespirometer

Oxygen consumption was analyzed in 2 mL glass chambers at 37°C using the Oroboros Oxygraph-2K high-resolution respirometer (Oroboros Instruments). The oxygen flux normalized on the cell number is calculated as the negative time derivative of the oxygen concentration, measured in sealed chambers, and normalized on the instrumental background (measured in a dedicated experiment before assaying the cells). RNT cells were treated with LN (100 µM) for 24 hours. The day of the respirometric analysis, 2 ×10^6^ cells resuspended in complete culture medium were introduced into the chambers and the basal respiratory activity was measured as routine respiration (R). The LEAK state (L) represents the non-phosphorylating state of uncoupled respiration due to proton leak, after the inhibition of ATP synthase by Oligo administration (5 nM) (Sigma-Aldrich, 1404-19-9). The capability of the electron transfer system (ETS) was measured by uncoupler titrations using the uncoupler Carbonyl Cyanide 3-ChloroPhenylhydrazone (CCCP, 1.5 μM/titration steps) (Sigma-Aldrich, C2759) as the readout of the maximal capacity of oxygen utilization (E). The residual oxygen consumption (ROX) that remains after the inhibition of ETS was determined by AA injection (2.5 μM) (Sigma-Aldrich, A8674). Data acquisition and analysis were performed using DatLab software (Oroboros Instrument) and the oxygen fluxes recorded in the individual titration steps were corrected for ROX.

### Statistical analysis

All data were expressed as the mean ± standard deviation (SD) or the mean ± standard error (SEM). Differences between groups were compared using Student’s *t*-test or ANOVA following post-hoc correction, with the use of Prism 10 (GraphPad Software). Statistical significance was defined when P < 0.05. P-values are reported only when biologically relevant, as indicated in Figure Legends. When differences were not statistically significant or the comparison was not biologically relevant, no indication was reported in the Figures.

## Results

### LN induces hepatocyte proliferation in vitro

We have previously reported that, in vivo, lead nitrate (LN) induces proliferation of liver cells [12]. However, it remains unclear whether LN exerts its mitogenic effect directly on hepatocytes or whether this effect is mediated by non-parenchymal cells, including macrophages and Kupffer cells, suggesting a non-cell-autonomous mechanism [14–16].

**Fig. 1:**
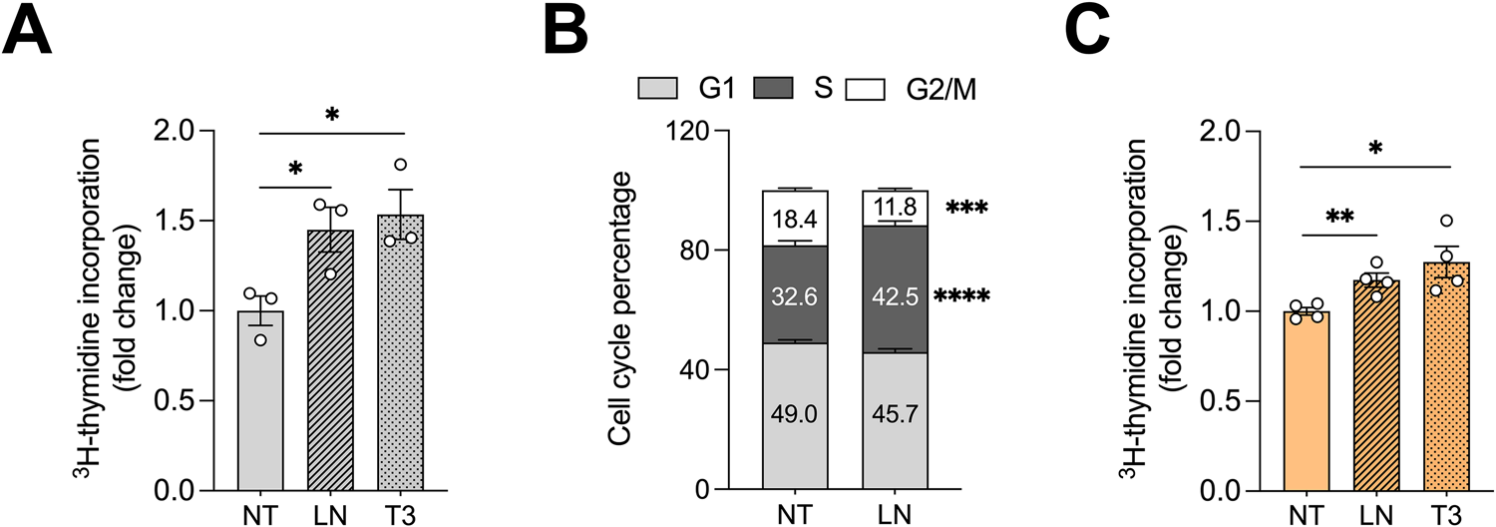
LN induces hepatocyte proliferation in vitro*.* **A** RNT cells were treated with LN (100 µM) or T3 (100 nM) for 24 hours and incubated for the last 6 hours in the presence of [^3^H]-thymidine to assess cell proliferation. Data are expressed as CPM and presented as fold change relative to the NT condition. Data are represented as mean ± SEM. Each dot represents a biological replicate, n = 3. Student *t*-test, *P < 0.05; **B** NT and LN-treated RNT cells were stained with PI (50 µg/mL) to assess the cell cycle. FACS analysis was performed to determine cell distribution in different cell cycle phases (G1, S, and G2/M). The percentage of cells in each phase was quantified using FlowJo software. Data are represented as mean ± SEM, n = 4. Two-way ANOVA followed by Tukey’s correction, ***P < 0.001, ****P < 0.0001; **C** THLE-2 cells were treated with LN (100 µM) or T3 (100 nM) for 48 hours and incubated for the last 24 hours in the presence of [^3^H]-thymidine to assess cell proliferation. Data are expressed as CPM and presented as fold change relative to the NT condition. Data are represented as mean ± SEM. Each dot represents a biological replicate, *n* = 4. Student *t*-test, *P < 0.05, **P < 0.01. Abbreviations: CPM, counts per minute; FACS, cytofluorimetric analysis; LN, lead nitrate; NT, untreated; PI, Propidium Iodide; T3, triiodothyronine.

To this aim, we measured LN-induced proliferation in two hepatocyte models – rat immortalized non-tumorigenic (RNT) and human immortalized non-tumorigenic (THLE-2) hepatocytes. LN exposure induced a marked increase in the proliferation of RNT cells, as evidenced by a significant increase in radiolabeled [^3^H]-thymidine incorporation (Fig. 1A) and in the S-phase fraction of cell cycle (Fig. 1B). The ability of LN to promote proliferation is not restricted to rat hepatocytes since it was also observed in human THLE-2 cells (Fig. 1C).

Moreover, the mitogenic effect of LN was comparable to that induced by triiodothyronine (T3), a known hepatic mitogen, used as a positive control in both cell lines (Fig. 1A, C). These results demonstrate that LN exerts a direct proliferative stimulus on immortalized non-tumorigenic hepatocytes.

### LN-induced cell proliferation is associated with the activation of the KEAP1-NRF2 pathway

The critical role of nuclear factor (erythroid-derived 2)-like 2 (NRF2) in LN-induced hepatocyte proliferation in vivo has been evidenced by the inability of *Nrf2*-KO rats to respond to the mitogenic effect of the metal [11]. Nevertheless, it remains unclear whether the activation of the Kelch-like ECH-associated protein 1 (KEAP1)-NRF2 pathway is either a direct effect of LN on hepatocytes or it occurs in non-parenchymal cells.

**Fig. 2:**
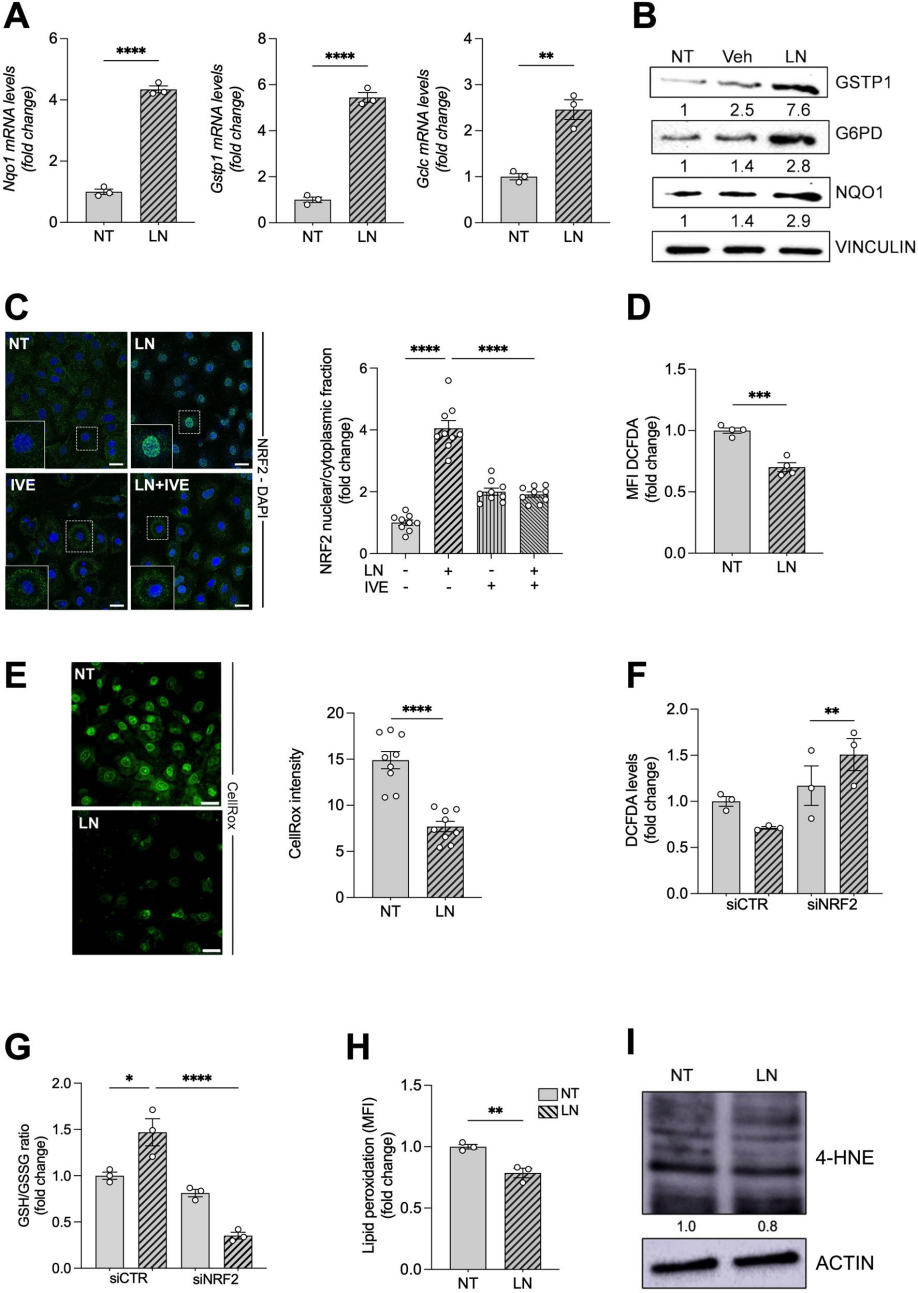
LN induces the expression of NRF2 target genes and reduces ROS generation in RNT cells. **A** The RNA derived from RNT cells either NT or treated for 24 h with LN (100 µM) was analyzed by qRT-PCR analysis using the assay described in the figure. Relative gene expression was calculated using *β-actin* as endogenous control. Data are presented as fold change relative to the NT condition. Data are represented as mean ± SEM. Each dot represents a biological replicate, n = 3. Student *t*-test. **P < 0.01, and ****P < 0.0001; **B** Western blot analysis of GSTP1, G6PD, and NQO1 protein levels was performed in NT, Veh conditions, and LN-treated RNT cells. Vinculin was used as loading control. Western blot quantification of 3 biological replicates was calculated using ImageJ software; **C** Immunofluorescence staining of NRF2 was performed in RNT cells NT or treated with LN (100 µM) with or without IVE (5 µM) for 24 hours. Representative confocal images of stained cells (left) are shown (green: NRF2; blue: DAPI, nuclei. Scale bar, 25 µm – inset of a higher magnification cell). Quantification of NRF2 nuclear/cytoplasmic signal fraction (right) is reported. Data are presented as fold change relative to the NT condition. Data are represented as mean ± SEM. Each dot represents a replicate: three biological replicates were performed in technical triplicate, n = 9. One-way ANOVA followed by Tukey’s correction, ****P < 0.0001. **D** RNT cells were treated with LN (100 µM) for 24 hours and subjected to FACS analysis to measure ROS levels with DCFDA fluorescent probe. Data are presented as fold change relative to the NT condition. Data are represented as mean ± SEM. Each dot represents a biological replicate, n = 4. Student *t*-test, ***P < 0.001; **E** RNT cells were treated with LN (100 µM) for 24 hours and subjected to confocal analysis. Representative pictures of CellRox-stained cells are shown (Green: CellRox. Scale bar, 25 µm). Quantification of CellRox is reported. Data are represented as mean ± SEM. Each dot represents a replicate: three biological replicates were performed in technical triplicate, n = 9. Student *t*-test, ****P < 0.0001; **F** ROS levels were measured using DCFDA probe in siCTR- and siNRF2-transfected RNT cells NT or treated with LN (100 µM) for 24 hours. Data are presented as fold change relative to the NT condition of siCTR. Data are represented as mean ± SEM. Each dot represents a biological replicate, n = 3. Two-way ANOVA followed by Tukey’s correction, **P < 0.01; **G** The GSH/GSSG levels ratio was measured in siCTR- and siNRF2-transfected RNT cells NT or treated with LN (100 µM) for 24 hours. Data are presented as fold change relative to the NT condition of siCTR. Data are represented as mean ± SEM. Each dot represents a biological replicate, n = 3. One-way ANOVA followed by Tukey’s correction, *P < 0.05, ****P < 0.0001; **H**, **I** RNT cells were treated with LN (100 µM) for 24 hours and subjected to FACS using BODIPY^581/591^-C_11_, a proxy of lipid peroxidation **H**, and western blot analyses to assess the levels of 4-HNE **I**. Actin was used as loading control. Western blot quantification of 3 biological replicates was calculated using ImageJ software. Data are presented as fold change relative to the NT condition. Data are represented as mean ± SEM. Each dot represents a biological replicate, n = 3. Student *t*-test, **P < 0.01. Abbreviations: *β-actin*: beta-actin; 4HNE, 4-hydroxynonenal; DCFDA, 5,6-Carboxy-2’,7’-Dichlorofluorescein Diacetate; FACS, cytofluorimetric analysis; GCLC/*Gclc*, glutamate-cysteine ligase catalytic subunit; GSH/GSSG, reduced glutathione/oxidized glutathione; GSTP1/*Gstp1*, placental glutathione S-transferase; IVE, ivermectin; LN, lead nitrate; MFI, mean fluorescent intensity; NQO1/*Nqo1*, NAD(P)H quinone dehydrogenase 1; NRF2, nuclear factor (erythroid-derived 2)-like 2; NT, untreated; Veh, vehicle.

As shown in Fig. 2A and B, LN significantly increased the expression of several NRF2 target genes including *Nqo1, Gstp1, Gclc, and G6pd*, at mRNA or protein level in RNT hepatocytes. Tumor necrosis factor-alpha (TNF-α), which is linked to NRF2 signaling and can be produced by hepatocytes under inflammatory/pathological conditions [22, 23], did not contribute to this response: *Tnf-α* mRNA levels were undetectable in both untreated and LN-exposed cells, indicating that the effect was TNF-α–independent. Since activation of the KEAP1-NRF2 pathway requires NRF2 translocation into the nucleus, we performed nuclear and cytoplasmic fractionation and subsequent NRF2 immunoblotting (Supplemental Fig. S1), together with confocal microscopy analysis (Fig. 2C). Notably, we observed a marked nuclear accumulation after LN treatment. Inhibition of importin‑mediated transport using ivermectin (IVE) prevented this translocation, as evidenced by a reduced nuclear‑to‑cytoplasmic NRF2 ratio (Fig. 2C).

Notably, T3-induced RNT cells proliferation did not result in the activation of the KEAP1-NRF2 pathway (Supplemental Fig. S2).

### ROS generation is not involved in NRF2 activation

Since an increase in reactive oxygen species (ROS) leads to the disruption of the KEAP1-NRF2 interaction and to NRF2 nuclear translocation [24], we investigated whether LN-induced ROS levels could be responsible for the activation of the KEAP1-NRF2 pathway. As shown, rather than increased ROS levels, LN-treated cells exhibited altered redox homeostasis, with reduced levels of total ROS quantified using DCFDA probes by FACS analysis (Fig. 2D). ROS levels were further analyzed with CellROX staining and quantified by confocal microscopy (Fig. 2E). Since the reduced ROS levels observed 24 hours after LN could be the consequence of a compensatory mechanism caused by the activation of the KEAP1-NRF2 pathway, ROS levels were also measured at earlier times after LN. However, no increase in ROS levels was observed even soon after LN treatment in RNT cells (10 minutes to 6 hours, Supplemental Figure S3).

Notably, NRF2 silencing led to increased baseline ROS levels, consistent with its role as a key regulator of antioxidant defense. Importantly, in siNRF2-transfected RNT cells, LN exposure failed to reduce ROS levels, in contrast to control cells (siCTR) (Fig. 2F). The observed reduction in ROS levels upon LN-exposure in siCTR-transfected cells was accompanied by a corresponding increase in the GSH/GSSG ratio, reflecting a higher proportion of reduced (GSH) to oxidized (GSSG) glutathione (Fig. 2G). This shift in the redox balance indicates an overall enhancement of the intracellular reducing environment, suggesting improved cellular antioxidant capacity. Accordingly, siNRF2-transfected RNT cells decreased the GSH/GSSG ratio despite LN administration, supporting the conclusion that LN-induced reduction in ROS is NRF2-dependent. This further confirms the key role of NRF2 in mediating the antioxidant response to LN (Fig. 2G).

In addition, we assessed lipid peroxidation, a key consequence of ROS-mediated damage to intracellular macromolecules, by measuring oxidized BODIPY^581/591^-C_11_ and 4-hydroxynonenal (4-HNE) protein adducts: LN treatment reduced the levels of lipid oxidation (Fig. 2H) and those of 4-HNE in RNT cells (Fig. 2I), thus indicating a general reduction of oxidative stress.

### NRF2 activation is mediated by LN-induced p62 accumulation

**Fig. 3:**
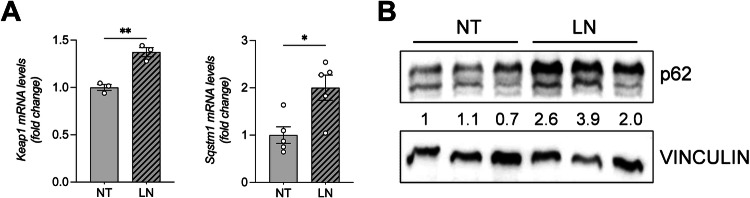
LN induces p62 accumulation in RNT cells. **A** The RNA derived from RNT cells either NT or treated for 24 hours with LN (100 µM) was analyzed by qRT-PCR analysis using the assay described in the figure. Relative mRNA expression was calculated using *β-actin* as endogenous control. Data are presented as fold change relative to the NT condition. Data are represented as mean ± SEM. Each dot represents a biological replicate, n = 3 for *Keap1* and n = 5 for *Sqstm1/p62*. Student *t*-test. *P < 0.05, **P < 0.01; **B** Western blot analysis of p62 protein level was performed in RNT cells 24 hours after LN (100 µM) treatment. Vinculin was used as loading control. Western blot quantification of 3 biological replicates was calculated using ImageJ software. Abbreviations: *β-actin*: beta-actin; *Keap1*, Kelch-like ECH-associated protein 1; LN, lead nitrate; NT, untreated; *Sqstm1*/p62, sequestosome 1.

Among various alternative mechanisms underlying NRF2 activation, epigenetic modulation of the *Keap1* promoter has been identified as a contributor to pathway activation [25]. To investigate whether decreased *Keap1* expression could explain the activation of the KEAP1-NRF2 pathway observed after LN treatment, we evaluated the expression of *Keap1* mRNA. As shown in Fig. 3A, increased *Keap1* mRNA levels were found in LN-treated when compared to untreated RNT cells, suggesting that LN-induced NRF2 nuclear translocation and transcriptional activation are not due to decreased *Keap1* levels.

Aberrant accumulation of the selective autophagy adaptor p62, also known as sequestosome 1 (p62/SQSTM1) [26], is known to disrupt the interaction between NRF2 and its inhibitor KEAP1 [27]. In fact, the KEAP1-interacting region of p62 binds to KEAP1 in a manner similar to the ETGE motif of NRF2, thereby preventing KEAP1 from trapping NRF2 and resulting in NRF2 stabilization and activation [27].

As shown in Fig. 3A, B, in LN-treated RNT cells we observed increased mRNA and protein levels of *Sqstm1*/p62, compared to untreated cells. Thus, accumulation of p62 appears to be responsible for the increased NRF2 accumulation, its nuclear translocation and transcriptional activation of antioxidant target genes shown in Fig. 2A–C.

### LN induces metabolic reprogramming in rat hepatocytes

**Fig. 4:**
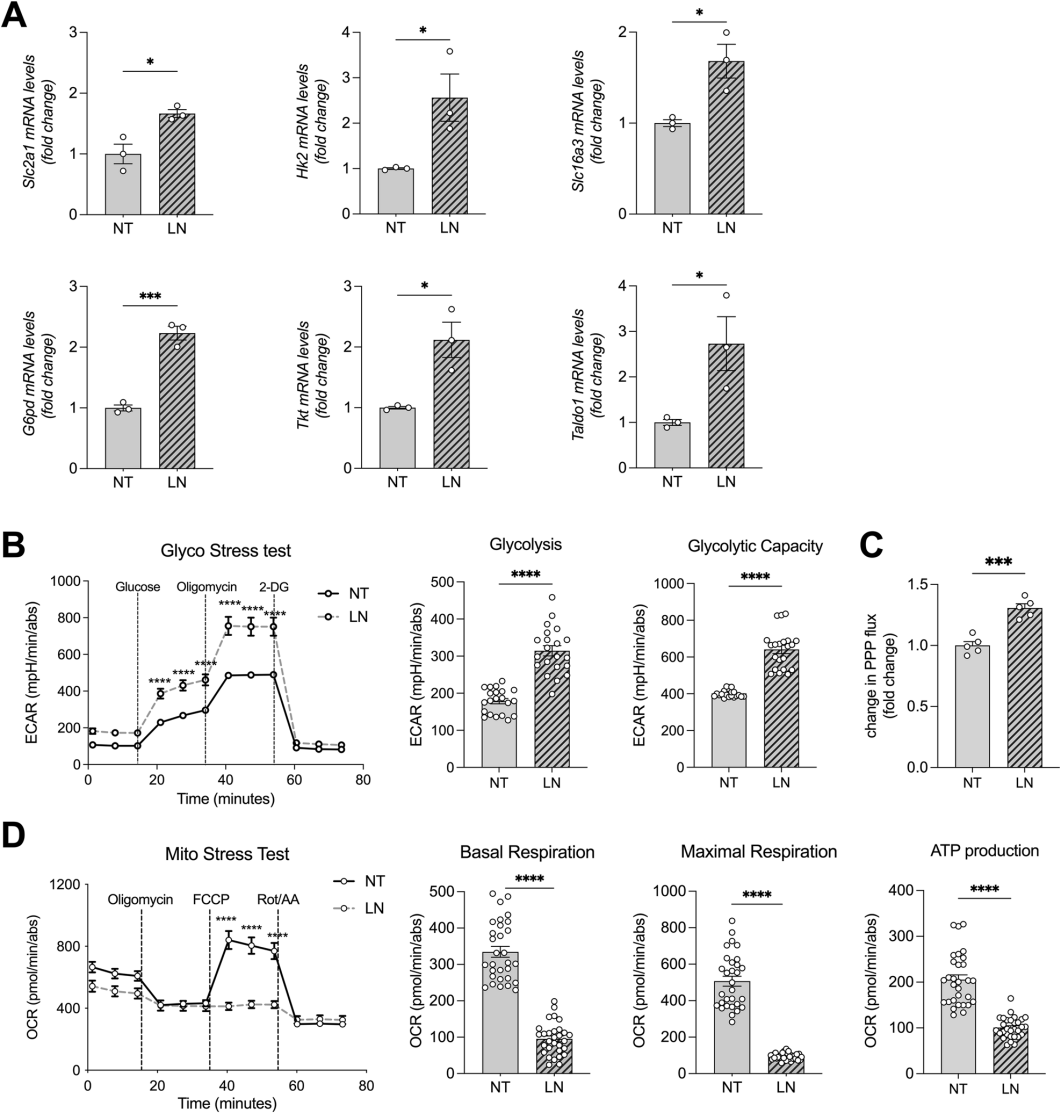
LN promotes metabolic reprogramming favoring glycolysis and PPP. **A** The RNA derived from RNT cells either NT or treated for 24 hours with LN (100 µM) was analyzed by qRT-PCR analysis using the assay described in the figure. Relative mRNA expression was calculated using *β-actin* as endogenous control. Data are presented as fold change relative to the NT condition. Data are represented as mean ± SEM. Each dot represents a biological replicate, n = 3. Student *t*-test. *P < 0.05, ***P < 0.001; **B** Left: Seahorse XFe96 Glycolysis Stress Test was performed on RNT cells cultured in the presence and absence of LN (100 µM) in RPMI medium without glucose, and ECAR was calculated in real-time after the administration of a saturating concentration of glucose (10 mM), the ATP synthase inhibitor oligomycin (1.5 µM), and the glycolysis inhibitor 2-DG (50 µM). Right: Glycolysis and glycolytic capacity were calculated as described in the Materials and Methods section and normalized on protein content. NT condition was used as comparator in the statistical analysis. Data are represented as mean ± SEM. One representative biological replicate is shown. Three biological replicates were performed in 7 technical replicates. Two-way ANOVA followed by Sidak’s correction (left) and Unpaired *t*-test with Welch’s correction (right). ****P < 0.0001; **C** RNT cells were treated with LN (100 µM) for 24 h and incubated for 1 h in the presence of radioactive glucose labeled in position 1 [1-^14^C] or in position 6 [6-^14^C]. Data are expressed as CPM and presented as fold change relative to the NT condition. Data are represented as mean ± SEM. Each dot represents a biological replicate, *n* = 5. Student *t*-test, ***P < 0.001; **D** Left: Seahorse XFe96 Mito Stress Test on LN-treated RNT cells. OCR was calculated in real-time after administration of ATP synthase inhibitor oligomycin (1.5 µM), FCCP (1 µM), and respiratory complex I inhibitor Rot together with respiratory complex III inhibitor AA (Rot/AA, 0.5 µM). Right: Basal and maximal respiration, and ATP production were calculated as described in the Materials and Methods section and normalized on protein content. NT condition was used as comparator in the statistical analysis. Data are represented as mean ± SEM. One representative biological replicate is shown. Three biological replicates were performed in 7 technical replicates. Two-way ANOVA followed by Tukey’s correction (left) and Student *t*-test (right), ****P < 0.0001. Abbreviations: 2-DG, 2-deoxy-glucose; CPM, counts per minute; ECAR, extracellular acidification rate; FCCP, proton uncoupler carbonyl cyanide p-triflouromethoxyphenylhydrazone; *G6pd*; glucose-6-phosphate dehydrogenase; *Hk2*, hexokinase 2; LN, lead nitrate; NT, untreated; OCR, oxygen consumption rate; Rot/AA, rotenone/antimycin A; *Slc2a1*, Solute carrier family 2 member 1; *Slc16a3*, Solute carrier family 16 member 3; *Taldo1*, transaldolase 1; *Tkt*, transketolase.

We have previously reported that LN treatment induced significant metabolic changes in hepatocytes, similar to those observed in preneoplastic lesions that progress to hepatocellular carcinoma (HCC) [11]. Specifically, LN exposure of RNT cells led to the upregulation of genes related to glycolysis (*Slc2a1, Hk2, Slc16a3* also known as *Glut1, Hk2, Mct4*, respectively*)* and pentose phosphate pathway (PPP) (*G6pd, Tkt, Taldo1*) (Fig. 4A). These transcriptional changes were supported by functional metabolic analyses. In RNT cells, Seahorse analysis (Fig. 4B and Supplemental Figure S4A) revealed enhanced glycolysis, glycolytic capacity and compensatory glycolysis following LN administration in RNT cells. Moreover, PPP flux analysis using radiolabeled [1-¹⁴C]- and [6-¹⁴C]-glucose tracing revealed increased flux in LN-treated RNT cells, further supporting a rerouting of glucose metabolism upon LN administration (Fig. 4C). This LN-induced metabolic reprogramming was further supported by the observation of impaired mitochondrial electron transport chain (ETS) activity and oxidative metabolism, both at baseline and under stress conditions (i.e., FCCP/CCCP injections, respectively). These findings, obtained through Seahorse and Oroboros oxygraphy-2K high-resolution respirometer-based assays indicated a reduction in mitochondrial ATP production following LN treatment in RNT cells (Fig. 4D and Supplemental Figure S4B). Crucially, the decrease in oxidative metabolism was not due to mitochondrial damage, as assessment of proton leak (Supplementary Figure S4B, leak), mitochondrial mass (MitoTracker Green fluorescence intensity) and mitochondrial morphology (aspect ratio and form factor) (Supplemental Figure S4C) showed no significant alteration.

Notably, T3 did not induce metabolic reprogramming in RNT cells as supported by qRT-PCR analysis of metabolic-related genes (Supplemental Figure S2) and functional metabolic analysis using an Oroboros-based assay (Supplemental Figure S5). This confirms a unique effect exerted by LN that could not be translated to other established mitogenic stimuli. Krueppel-like factor 9 (*Klf9*), a bona fide T3/thyroid hormone receptor target gene [28], was used as a positive control to verify effective T3 delivery and receptor activation in RNT cells (Supplemental Figure S2).

### NRF2 silencing abrogates LN-induced metabolic reprogramming

**Fig. 5:**
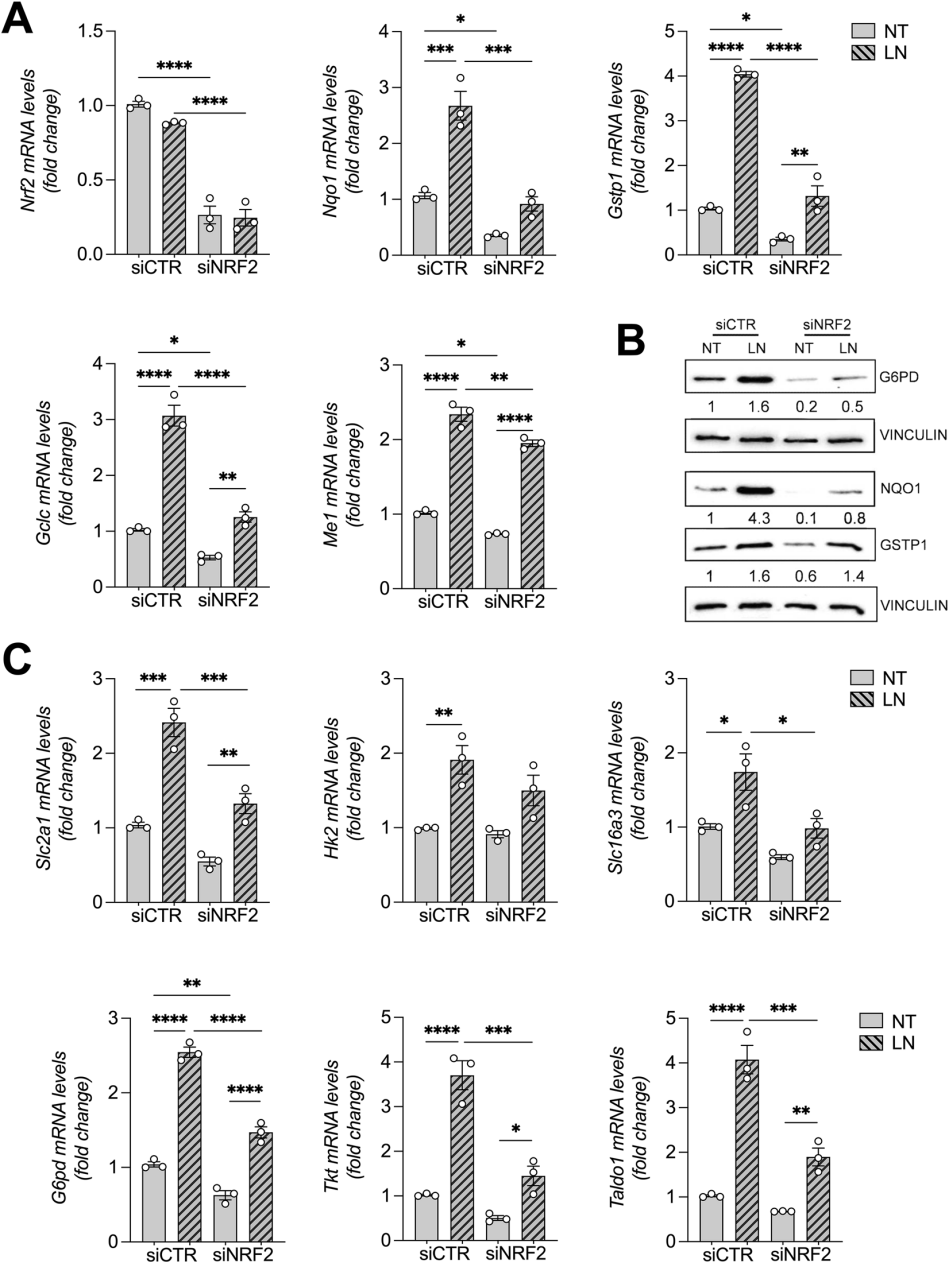
NRF2 silencing abrogates LN-induced metabolic reprogramming of RNT cells. **A** The RNA derived from siCTR- or siNRF2-transfected RNT cells either NT or treated for 24 hours with LN (100 µM) was analyzed by qRT-PCR analysis using the assay described in the figure. Relative mRNA expression was calculated using *β-actin* as endogenous control. Data are presented as fold change relative to the NT condition of siCTR. Data are represented as mean ± SEM. Each dot represents a biological replicate, *n* = 3. One-way ANOVA with Tukey’s correction. *P < 0.05, **P < 0.01, ***P < 0.001, ****P < 0.0001; **B** Western blot analysis of G6PD, NQO1, and GSTP1 protein levels was performed in siCTR- and siNRF2- transfected RNT cells treated with LN (100 µM) for 24 hours. Vinculin was used as loading control. Western blot quantification of 3 biological replicates was calculated using ImageJ software; **C** The RNA derived from siCTR- or siNRF2-transfected RNT cells either NT or treated for 24 hours with LN (100 µM) was analyzed by qRT-PCR analysis using the assay described in the figure. Relative mRNA expression was calculated using *β-actin* as endogenous control. Data are presented as fold change relative to the NT condition of siCTR. Data are represented as mean ± SEM. Each dot represents a biological replicate, *n* = 3. One-way ANOVA followed by Tukey correction. *P < 0.05, **P < 0.01, ***P < 0.001, ****P < 0.0001. Abbreviations: *Gclc*, glutamate-cysteine ligase catalytic subunit; *Gstp1*/GSTP1, placental glutathione S-transferase; *G6pd*/G6PD; glucose-6-phosphate dehydrogenase; *Hk2*, hexokinase 2; LN, lead nitrate; *Me1*, malic enzyme; *Nqo1*/NQO1, NAD(P)H quinone dehydrogenase 1; *Nrf2*, nuclear factor (erythroid-derived 2)-like 2; NT, untreated; *Slc2a1*, Solute carrier family 2 member1; *Slc16a3*, Solute carrier family 16 member 3; *Taldo1*, transaldolase 1; *Tkt*, transketolase.

To further establish the role of NRF2 in LN metabolic reprogramming, we performed silencing experiments in RNT cells. qRT-PCR analysis showed a significant inactivation of *Nrf2*, as demonstrated by reduced expression of its mRNA levels as well as of *Nrf2* target genes, such as *Nqo1, Gstp1, Gclc*, and *Me1* (Fig. 5A). Decreased expression of target genes was also observed at the protein level (Fig. 5B).

Next, we examined the effect of LN treatment on metabolic changes in RNT upon *Nrf2* silencing. As expected, *Nrf2* silencing strongly impaired LN-induced expression of *Nrf2* target genes and of genes involved in glycolysis (*Slc2a1, Hk2, Slc16a3*) as well as in the PPP pathways (*G6pd, Tkt, Taldo1*) (Fig. 5C), thus preventing the metabolic reprogramming observed in siCTR RNT cells.

As demonstrated in Fig. 2F, *Nrf2* silencing abrogated the cell-autonomous effect of LN on redox homeostasis in RNT cells. Moreover, *Nrf2* silencing reversed the LN-induced alterations in both basal (routine) and maximal (E) respiration, resulting in a generalized increase in oxygen consumption that appeared independent of LN exposure (Supplemental Figure S6).

**Fig. 6:**
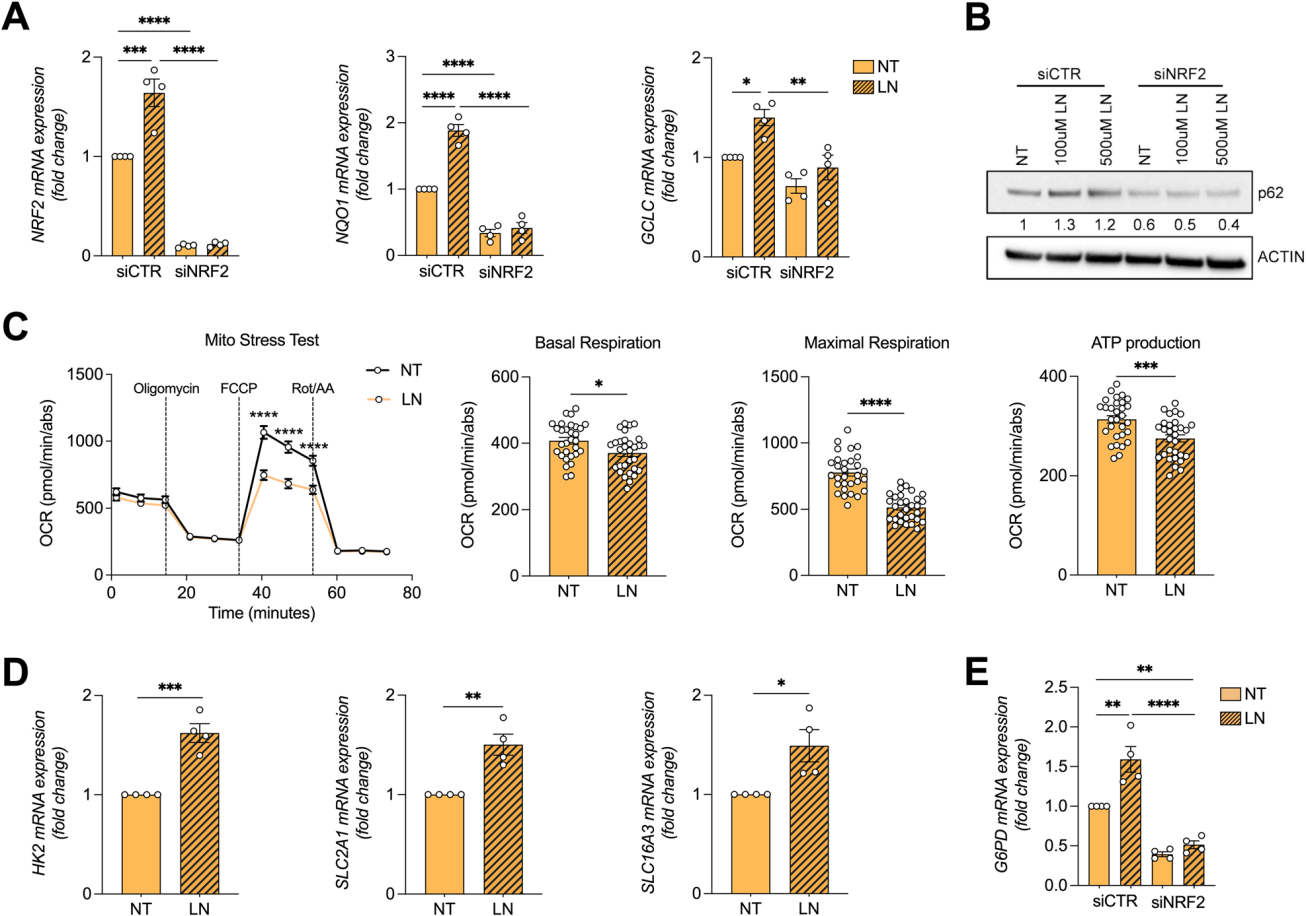
LN induces NRF2 activation and metabolic reprogramming also in THLE-2 human hepatocytes. **A** The RNA derived from siCTR- or siNRF2-transfected THLE-2 cells either NT or treated for 24 hours with LN (500 µM) was analyzed by qRT-PCR analysis using the assay described in the Figure. Relative mRNA expression was calculated using *β-actin* as endogenous control. Data are presented as fold change relative to the NT condition of siCTR. Data are represented as mean ± SEM. Each dot represents a biological replicate, n = 4. One-way ANOVA followed by Tukey’s correction. *P < 0.05, **P < 0.01, ***P < 0.001, ****P < 0.0001; **B** Western blot analysis of p62 protein level was performed in siCTR- and siNRF2-transfected THLE-2 cells treated with LN (100 and 500 µM) for 24 hours. β-ACTIN was used as loading control. Western blot quantification of 3 biological replicates was calculated using ImageJ software; **C** Left: Seahorse XFe96 Mito Stress Test on LN-treated THLE-2 cells (100 µM for 24 hours). OCR was calculated in real-time after administration of ATP synthase inhibitor Oligo (1.5 µM), FCCP (1 µM), and respiratory complex I inhibitor Rot together with respiratory complex III inhibitor AA (Rot/AA, 0.5 µM). Right: Basal and maximal respiration, and ATP production were calculated as described in the Materials and Methods section and normalized on protein content. NT condition was used as comparator in the statistical analysis. Data are represented as mean ± SEM. One representative biological replicate is shown. Three biological replicates were performed in 7 technical replicates. Two-way ANOVA followed by Tukey’s correction (left) and Student *t*-test (right), *P < 0.05, ***P < 0.001; ****P < 0.0001; **D** The RNA derived from THLE-2 cells either NT or treated for 24 hours with LN (500 µM) was analyzed by qRT-PCR analysis using the assay described in the figure. Relative mRNA expression was calculated using *β-actin* as endogenous control. Data are presented as fold change relative to the NT condition. Data are represented as mean ± SEM. Each dot represents a biological replicate, n = 4. Student *t*-test. *P < 0.05, **P < 0.01, ***P < 0.001; **E** The RNA derived from siCTR- or siNRF2-transfected THLE-2 cells either NT or treated for 24 hours with LN (500 µM) was analyzed by qRT-PCR analysis using the assay described in the figure. Relative mRNA expression was calculated using *β-actin* as an endogenous control. Data are presented as fold change relative to the NT condition of siCTR. Data are represented as mean ± SEM. Each dot represents a biological replicate, n = 4. One-way ANOVA followed by Tukey’s correction. **P < 0.01, ****P < 0.0001. Abbreviations: *β-actin*: beta-actin; FCCP, proton uncoupler carbonyl cyanide p-triflouromethoxyphenylhydrazone; G6PD; glucose-6-phosphate dehydrogenase; GCLC, glutamate-cysteine ligase catalytic subunit; HK2, hexokinase 2; LN, lead nitrate; NQO1, NAD(P)H quinone dehydrogenase 1; NRF2, nuclear factor (erythroid-derived 2)-like 2; NT, untreated; Oligo, oligomycin; OCR, oxygen consumption rate; Rot/AA, rotenone/antimycin A; SLC16A1, Solute carrier family 16 member 1; SLC16A3, Solute carrier family 16 member 3.

To investigate whether the NRF2-dependent metabolic reprogramming induced by LN could also occur in human hepatocytes, we performed a series of experiments in human THLE-2 cells. As shown in Fig. 6A, a significant increase in mRNA levels of *NRF2* as well as *NQO1* and *GCLC*, established NRF2-target genes, was observed upon LN treatment. These effects were almost completely abrogated by *NRF2* silencing. Similar to what observed in rat RNT cells, LN induced p62 accumulation also in human THLE-2 hepatocytes (Fig. 6B). As expected, *NRF2* silencing abrogated LN-induced accumulation of p62 on THLE-2 cells (Fig. 6B).

Furthermore, LN induced metabolic reprogramming also in human THLE-2 hepatocytes, as shown by a reduction in both basal and maximal oxidative metabolism, as well as mitochondrial ATP production (Fig. 6C). Accordingly, an upregulation of glycolytic genes, including *HK2, SLC2A1 (GLUT1), and SLC16A3 (MCT4)*, was observed *(*Fig. 6D), along with increased expression G6PD (Fig. 6E).

Overall, these results demonstrate that the mitogenic effect of LN on hepatocytes, as well as the induction of KEAP1-NRF2 pathway activation and the metabolic reprogramming, are cell-autonomous. In addition, our findings also suggest that NRF2 activation is essential for metabolic reprogramming induced by LN, but it is not shared by all the hepatic mitogens.

## Discussion

A complex metabolic rewiring, characterized by enhanced glucose and glutamine utilization and a decrease in oxidative phosphorylation (OXPHOS), has been reported in several cancer types [1, 2, 29, 30]. Although this metabolic switch has been well-documented in tumors, including hepatocellular carcinoma (HCC), it emerged that a profound deregulation of cellular energy metabolism also occurs in non-cancerous contexts, such as in differentiated proliferating cells, involved in pluripotency, immunity or angiogenesis [31]. A key feature observed in cells exhibiting the phenomenon of aerobic glycolysis is “rapid proliferation” [32]. Recently, Kowalik et al. [11] demonstrated that well-established features of cancer metabolic reprogramming, including enhanced glycolysis, increased activity of the oxidative branch of the pentose phosphate pathway (PPP), increased nucleic acid synthesis, altered amino acid content, and down-regulation of OXPHOS, arise in differentiated proliferating hepatocytes. This occurs in vivo following exposure to LN, a primary mitogen capable of inducing hepatocyte entry into the cell cycle in the absence of previous liver cell damage [12, 33].

Differentiated hepatocytes can be induced to proliferate not only in response to cell loss (compensatory regeneration), but also following treatment with various compounds known as primary mitogens (direct hyperplasia), such as LN, triiodothyronine (T3), peroxisome proliferators, or 9-*cis* retinoic acid [33]. Liver cell growth triggered by primary mitogens involves patterns of growth factor modulation and signal transduction that are distinct from those observed in compensatory regeneration, including the activation of NF-κB and immediate early genes such as *c-fos*, *c-jun*, and *egr-1*. Among hepatic mitogens, LN not only induces hepatocyte proliferation [12], but also elicits biochemical features characteristic of hepatic preneoplastic nodules [11, 34–36]. However, despite repeated waves of hepatocyte proliferation and associated metabolic alterations, LN does not promote cancer development, even after prolonged administration [37, 38].

Our findings are consistent with and build upon those of Kowalik et al. [11], demonstrating that LN alone is sufficient to induce a similar metabolic rewiring directly in vitro, in both rat and human non-tumorigenic hepatocyte lines, independent of non-parenchymal cells or inflammatory mediators. Notably, by comparing LN to another hepatocyte mitogen, T3, which promotes hepatocyte proliferation in vivo without activating NRF2, we reveal a novel mechanistic distinction: only LN, and not T3, triggers NRF2-mediated metabolic reprogramming.

In the present study, using rat and human non-tumorigenic immortalized cell lines displaying features of typical mature hepatic epithelial cells, we investigated whether (1) hepatocyte proliferation is directly driven by LN; (2) induction of metabolic reprogramming is a direct effect of LN exerted on hepatocytes.

The key findings of this study are as follows: (1) LN induces cell proliferation and metabolic reprogramming in normal rat and human hepatocytes; (2) LN induces p62-mediated NRF2 activation; (3) no metabolic reprogramming was detected in proliferating hepatocytes treated with T3, a liver mitogen unable to induce NRF2 activation; (4) NRF2 silencing abrogates LN-induced metabolic reprogramming.

With regard to hepatocyte proliferation, previous studies have reported that a single dose of LN can stimulate liver cell proliferation in vivo [12]. Moreover, the finding that LN-induced liver cell proliferation is accompanied by a rapid increase in hepatic expression of *TNF*-α mRNA [15, 16], and that pretreatment with dexamethasone or gadolinium chloride strongly impaired hepatocyte proliferation suggested that this cytokine may be involved in triggering LN-induced hepatocyte proliferation [15, 16, 39]. Our study demonstrates that LN exerts a direct proliferative effect on rat and human normal hepatocytes, which is *TNF*-α independent, without the involvement of non-parenchymal cells.

Exposure of RNT cells to LN led to the up-regulation of glycolysis-related genes and those involved in the PPP, while impairing the activity of the mitochondrial electron transport chain (ETS), consistent with metabolic reprogramming. These metabolic changes observed following LN treatment in RNT cells largely recapitulated those metabolic changes observed in human HCC [40], in preneoplastic and neoplastic lesions in the R-H model of hepatocarcinogenesis [11] and in vivo in normal hepatocytes exposed to LN [12]. The transcription factor NRF2, a master regulator of the cellular antioxidant response, plays a key role in regulating metabolic reprogramming and controlling the PPP flux [7]. Activation of NRF2 not only increases glucose uptake and directs it to the PPP by modulating the expression of enzymes in both the oxidative and non-oxidative branches of the PPP, such as *G6PD, TKT, TALDO1*, but also controls the expression of enzymes involved in the synthesis of NADPH [9]. Notably, the metabolic switch observed in LN-treated cells was associated with a strong activation of NRF2 target genes, such as *NQO1, GSTP1*, and *GCLC*. In a previous study designed to test the transcriptional activation capacity of LN and to identify metal-responsive promoters, Tully et al. reported a dose-dependent increase of the glutathione-S-transferase Ya subunit (GSTYa) gene promoter in HepG2 cells [41]. Increased activity of NRF2 was due to the accumulation of p62, which, beyond its canonical role in autophagy, emerged as a signaling hub capable of orchestrating oxidative stress and metabolic reprogramming [42]. Increased p62 competes with the DLG motif for binding to the KEAP1 DC pocket, preventing NRF2 binding and leading to NRF2 stabilization and the increased expression of cytoprotective genes [27]. In line with these studies, we herein demonstrated that the accumulation of p62 contributed to the increased activity of NRF2 in hepatocytes. However, further p62 loss-of-function studies would help strengthen the mechanistic link between p62, autophagy and NRF2.

Transient NRF2 activation may result in cytoprotective antioxidant responses by reducing ROS production and enhancing NADPH availability through G6PD. Consequently, in this study, *NRF2* silencing significantly reduced the LN-induced expression of NRF2 target genes, as well as genes involved in glycolysis and the PPP pathways, suggesting that NRF2 is essential for the metabolic reprogramming induced by LN in hepatocytes.

According to what observed in vivo in models of hepatocyte proliferation [12], exposure to T3 did not result in the activation of NRF2 target genes and metabolic reprogramming, suggesting that NRF2 activation is a *sine qua non* condition for triggering metabolic reprogramming of proliferating hepatocytes. To our knowledge, this is the first study to directly compare the metabolic effects of distinct mitogens in hepatocytes and to identify NRF2 activation as a key discriminant linking LN-induced proliferation to metabolic remodeling. An additional strength of this study is the establishment of a novel in vitro model for the study of the molecular mechanisms underlying metabolic changes in normal and neoplastic hepatocytes. The development of such models provides a valuable tool for evaluating responses to therapeutic strategies and improving reliability of preclinical research. In vitro models that closely mirror in vivo conditions, such as those involving metabolic deregulation, may enhance the predictive accuracy of the therapeutic efficacy in potential treatment approaches.

## Supplementary information

Supplemental Data

Uncropped blots

## Data Availability

Data will be made available from the corresponding authors upon request.
