# Supplementary material for: Lead induces cell-autonomous proliferation and metabolic reprogramming of hepatocytes
Source: Cell Death Dis. 2025 Nov 10;16(1):816. doi: 10.1038/s41419-025-08134-6 (PMC12603040; doi:10.1038/s41419-025-08134-6)
Supplement: Supplementary file 1 — Supplemental Data [file 41419_2025_8134_MOESM1_ESM.pdf]

SUPPLEMENTAL DATA

Lead induces cell autonomous proliferation and metabolic reprogramming of hepatocytes

Marina Serra, Alfredo Smiriglia, Cristina Migliore, Andrea Caddeo, Nicla Lorito, Gabriele Tani, Giorgia Zedda, Amedeo Columbano, Andrea Perra, Silvia Giordano, Marta Anna Kowalik*, and Andrea Morandi*

The Supplemental Data contains:
Supplementary Figures S1-S6 and Table S1 and S2

***Correspondence**:

andrea.morandi@unifi.it

ma.kowalik@unica.it

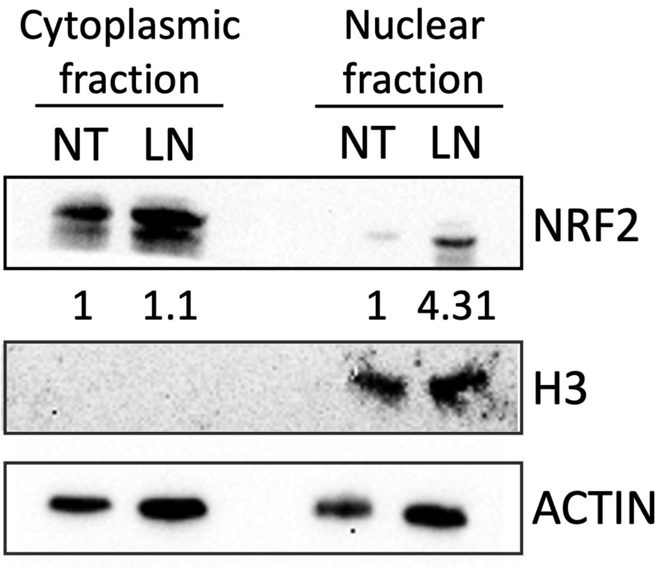

Supplemental Figure S1. NRF2 levels increases in the nuclear faction of RNT cells after LN administration. Western blot analysis of NRF2 protein levels was performed in NT and LN-treated RNT cells. Cytoplasmic and nuclear fractions were prepared as described in the Methods section. β-ACTIN was used as loading control. Histone H3 was used as a nuclear fraction marker. Western blot quantification was calculated using ImageJ software.

Abbreviations: LN, lead nitrate; NT, untreated; VINC, Vinculin.

Supplemental Figure S2

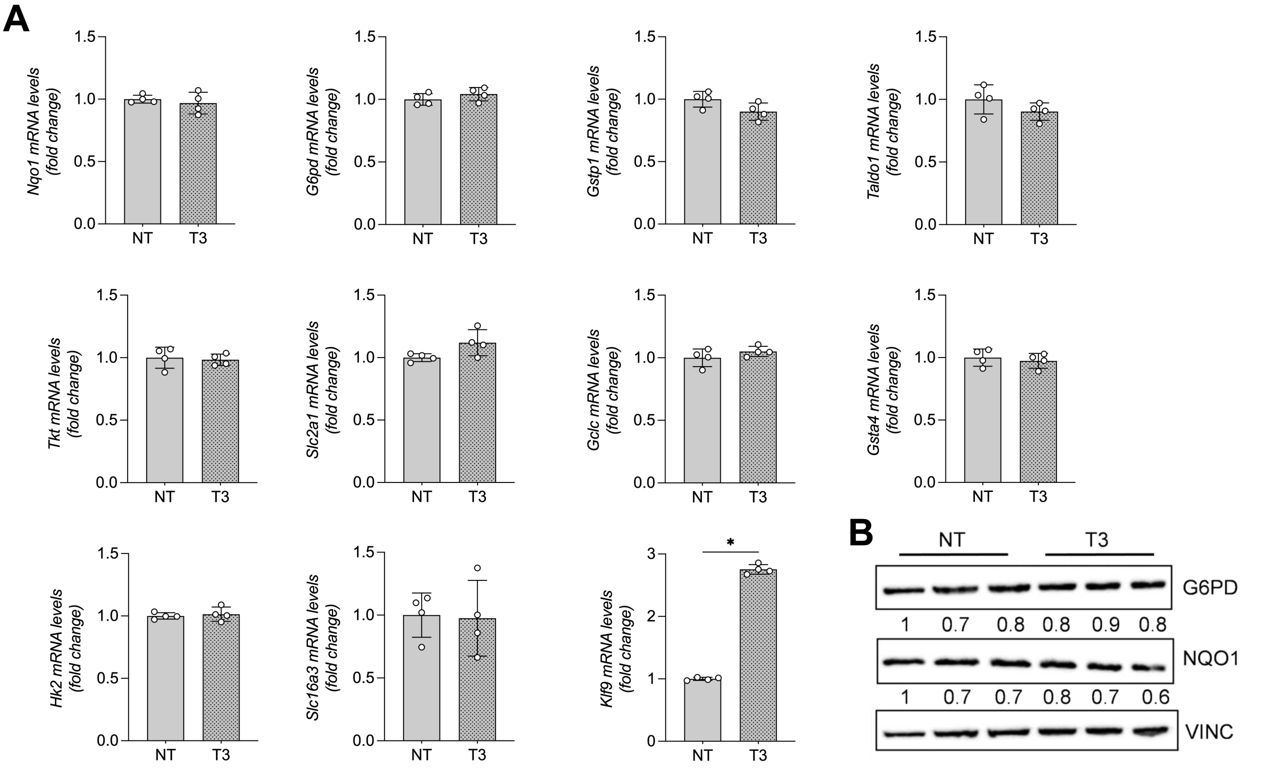

Supplemental Figure S2. No increased expression of NRF2 target genes or glycolytic genes is observed in T3-treated RNT cells. A) The RNA derived from RNT cells either NT or treated for 24 hours with T3 (100 µM) was analyzed by qRT-PCR analysis using the assay described in the figure. *Klf9*, a direct transcriptional target gene of T3 was used as a positive control of thyroid hormone receptor activation. Relative gene expression was calculated using *β-actin* as an endogenous control. Data are presented as fold change relative to the NT condition. Data are represented as mean ± SEM. Each dot represents a biological replicate, n = 4. Student *t*-test. *P <0.05; **B)** Western blot analysis of G6PD and NQO1 protein levels was performed in RNT cells treated with T3 (100 nM) for 24 hours. Vinculin was used as loading control. Western blot quantification of 3 biological replicates was calculated using ImageJ software.

Abbreviations: *G6pd*/G6PD, glucose-6-phosphate dehydrogenase; *Gclc*, glutamate-cysteine ligase catalytic subunit; *Gsta4*, glutathione S-Transferase Alpha 4; *Gstp1*, placental glutathione S-transferase; *Hk2*, hexokinase 2; *Klf9*, Kruppel-like factor 9; *Nqo1*/NQO1, NAD(P)H quinone dehydrogenase 1; NT, untreated; *Slc2a1*, Solute carrier family 2 member 1; *Slc16a3*, Solute carrier family 16 member 3; T3, triiodothyronine; *Tkt*, transketolase; *Taldo1*, transaldolase 1; VINC, Vinculin.

Supplemental Figure S3

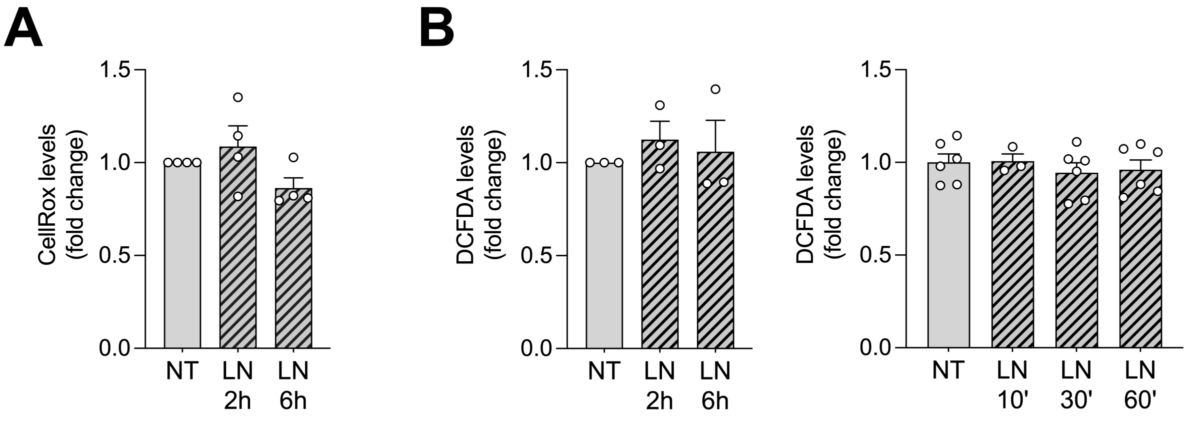

Supplemental Figure S3. No increased ROS generation is observed at early times after LN treatment in RNT cells. ROS levels were measured using CellRox (**A**) and DCFDA (**B**) fluorescent probes in LN-treated RNT cells at early time (from 10 minutes to 6 hours). Data are represented as mean ± SEM. Each dot represents a biological replicate, n ≥ 3. One-way ANOVA followed by Tukey's correction.

Abbreviations: DCFDA, 5,6-Carboxy-2',7'-Dichlorofluorescein Diacetate; LN, lead nitrate; NT, untreated.

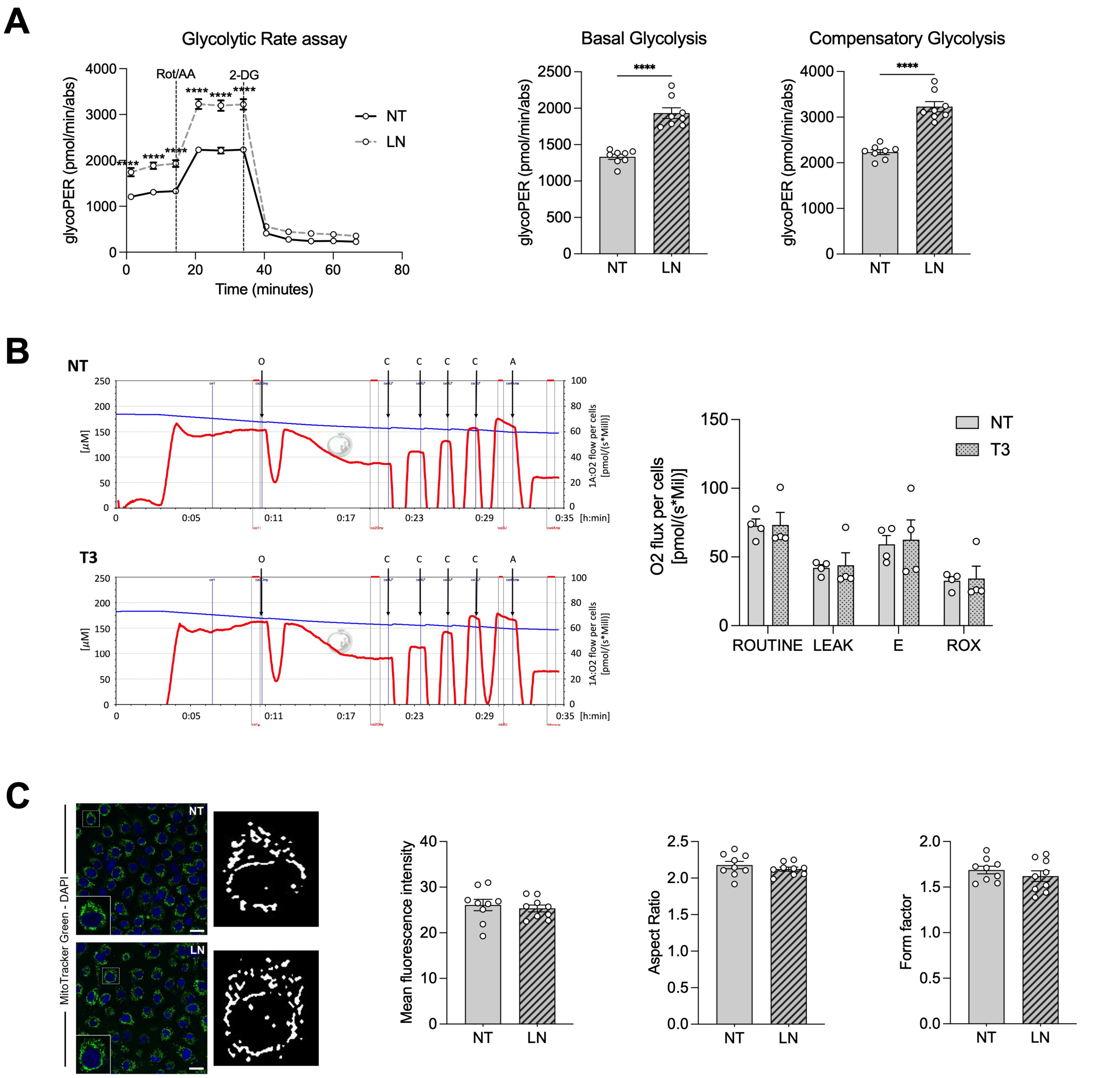

Supplemental Figure S4. LN induced metabolic reprogramming, without mitochondrial damage in RNT cells. A) Left: Seahorse XFe96 Glycolytic Rate Assay was performed on RNT cells cultured in the presence and absence of LN (100 µM) in a RPMI medium by concomitantly measuring the amount of OCR and ECAR after Rot/AA (0.5 µM) and 2-DG (50 mM) administration, thereby providing the GlycoPER. Right: Glycolysis and Compensatory Glycolysis were derived and normalized on protein content. NT condition was used as comparator in the statistical analysis. One representative biological replicate is shown. Three biological replicates were performed in 8 technical replicates. Two-way ANOVA followed by Sidak's correction (left) and Unpaired *t*-test (right), ****P < 0.0001. **B)** LN-treated RNT cells were subjected to high-resolution respirometry analysis by the Oroboros-O2K instrument. Left: Representative graphs of cell respirometry analysis in the

NT and treatment conditions. The blue curve represents the oxygen concentration, whereas the red slope shows the oxygen consumption before and after the serial injections of oligomycin (O), uncoupler CCCP (C), and Antimycin A (A). Right: Bar chart graph of basal oxygen consumption (Routine), proton leak (Leak), maximal oxygen consumption (E), and residual oxygen consumption (ROX) values in NT and LN treated cells. Data are represented as mean ± SEM. Each dot represents a biological replicate, n = 4. One-way ANOVA followed by Tukey's correction; **C)** RNT cells were treated with LN (100 μM) for 24 hours and subjected to confocal analysis. Left: Representative confocal images of MitoTracker Green-stained cells are shown with a higher magnification represented in the highlighted box (Green: MitoTracker Green; blue: DAPI. Scale bar, 25 μm). Right: Quantification of MitoTracker Green fluorescence intensity, mitochondrial morphology parameters (aspect ratio and form factor) were reported. Data are represented as mean ± SEM. Each dot represents a replicate: three biological replicates were performed in technical triplicate, n = 9. Abbreviations: 2-DG, 2-deoxy-glucose, A, Antimycin A; CCCP, Carbonyl cyanide m-chlorophenylhydrazone; ECAR, extracellular acidification rate; GlycoPER, Glycolytic Proton Efflux Rate; LN, lead nitrate; NT, untreated; O, oligomycin; OCR, oxygen consumption rate; ROX, residual oxygen consumption; Rot/AA, Rotenone/Antimycin A.

Supplemental Figure S5

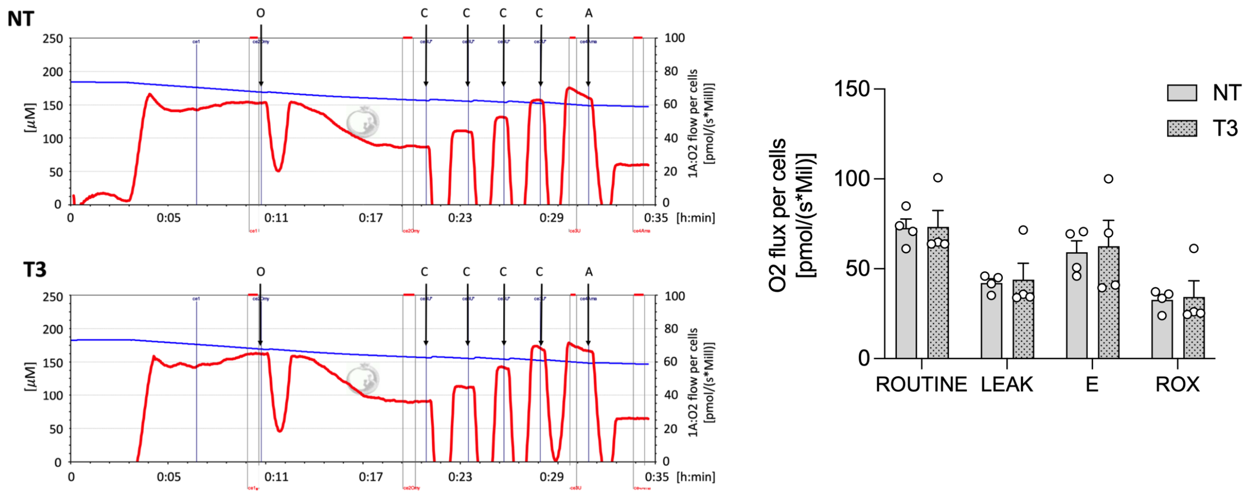

Supplemental Figure S5. No change in oxygen consumption occurs in T3-treated RNT cells. RNT cells treated with T3 (100 nM) for 24 hours were subjected to high-resolution respirometry analysis by the Oroboros-O2K instrument. Left: Representative graphs of cell respirometry analysis in NT and treatment conditions. The blue curve represents the oxygen concentration, whereas the red slope shows the oxygen consumption before and after the serial injections of oligomycin (O), uncoupler CCCP (C), and Antimycin A (A). Right: Bar chart graph of basal oxygen consumption (Routine), proton leak (Leak), maximal oxygen consumption (E), and residual oxygen consumption (ROX) values in NT and T3 treated cells. Data are represented as mean ± SEM. Each dot represents a biological replicate, n = 4. One-way ANOVA followed by Tukey's correction.

Abbreviations: A, Antimycin A; CCCP, Carbonyl cyanide m-chlorophenylhydrazone; NT, untreated; O, oligomycin; T3, triiodothyronine.

A

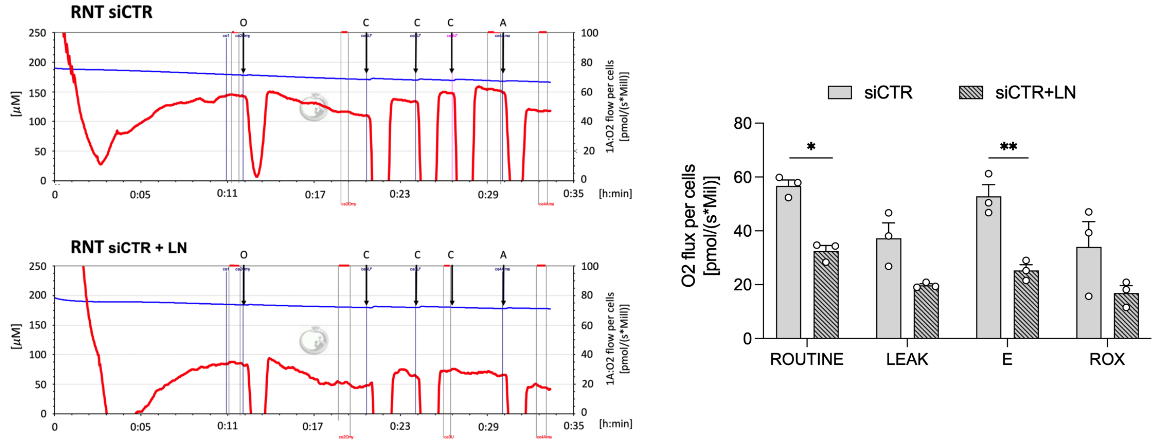

B

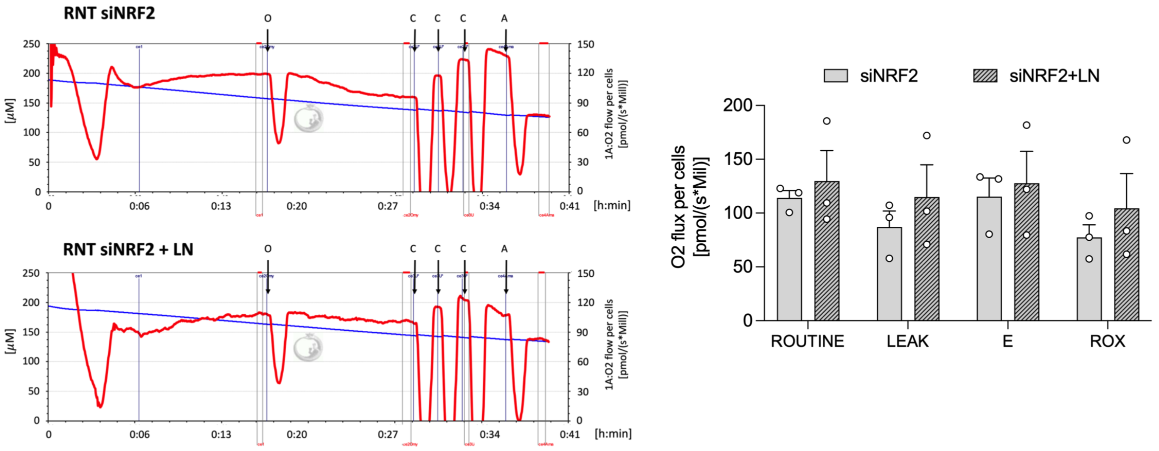

Supplemental Figure S6. NRF2 silencing abrogates LN-induced reduction in mitochondrial oxygen consumption in RNT cells. A) CTR and **B)** NRF2-silenced RNT cells treated with LN (100 µM) for 24 hours were subjected to high-resolution respirometry analysis by the Oroboros-O2K instrument. **A-B)** Top: Representative graphs of cell respirometry analysis in the NT and treatment conditions (LN). The blue curve represents the oxygen concentration, whereas the red slope shows the oxygen consumption before and after the serial injections of oligomycin (O), uncoupler CCCP (C), and Antimycin A (A). Bottom: Bar chart graph of basal oxygen consumption (Routine), proton leak (Leak), maximal oxygen consumption (E), and residual oxygen consumption (ROX) values in NT and LN treated cells silenced or not for NRF2. Data are represented as mean ± SEM. Each dot represents a biological replicate, n = 3. One-way ANOVA followed by Tukey's correction, *P < 0.05, **P < 0.01.

Abbreviations: A, Antimycin A; CCCP, Carbonyl cyanide m-chlorophenylhydrazone; LN, lead nitrate; NT, untreated; NRF2, nuclear factor (erythroid-derived 2)-like 2; O, oligomycin.

Supplemental Table S1. qPCR assays (Thermo Fisher Scientific)

Rat gene	**Assay ID**
Gclc	Rn00689048_m1
G6pd	Rn01529640_g1
Gstp1	Rn00821792_g1
Hk2	Rn00562457_m1
Keap1	Rn00589292_m1
Me1	Rn00561502_m1
Nfe2l2	Rn00477784_m1
Nqo1	Rn00566528_m1
Sqstm1	Rn00709977_m1
Slc2a1	Rn01417099_m1
Slc16a3	Rn00578115_m1
Taldo1	Rn00582620_m1
Tkt	Rn01453465_g1
Tnf	Rn99999017_m1

Human gene	**Assay ID**
GCLC	Hs00155249_m1
G6PD	Hs00166169_m1
HK2	Hs00606086_m1
NQO1	Hs01045993_g1
NFE2L2	Hs00231457_m1
Slc2a1	Hs00892681_m1
Slc16a3	Hs00358829_m1
TNF	Hs00174128_m1

Supplemental Table S2. List of antibodies

Antibody	Catalog number	Company	RRID
β-ACTIN	A3854	Sigma Aldrich	AB_262011
β-ACTIN	ab8227	Abcam	-
Vinculin	ab129002	Abcam	AB_11144129
4-HNE	bs-6313r	Bioss	-
GSTP	J311	MBL	-
G6PD	ab87230	Abcam	AB_1951900
Histone H3	MAB-91904	Immunological Sciences	-
NQO1	ab28947	Abcam	AB_881738
NRF2	16396-1-AP	Proteintech	AB_2782956
p62 (SQSTM1)	PM045	MBL	AB_1279301
Phospho-p62 (SQSTM1)	PM074	MBL	-
