# Supplementary material for: Lead induces cell-autonomous proliferation and metabolic reprogramming of hepatocytes
Source: Cell Death Dis. 2025 Nov 10;16(1):816. doi: 10.1038/s41419-025-08134-6 (PMC12603040; doi:10.1038/s41419-025-08134-6)
Supplement: Supplementary file 2 — Uncropped blots [file 41419_2025_8134_MOESM2_ESM.pdf]

Figure 2B

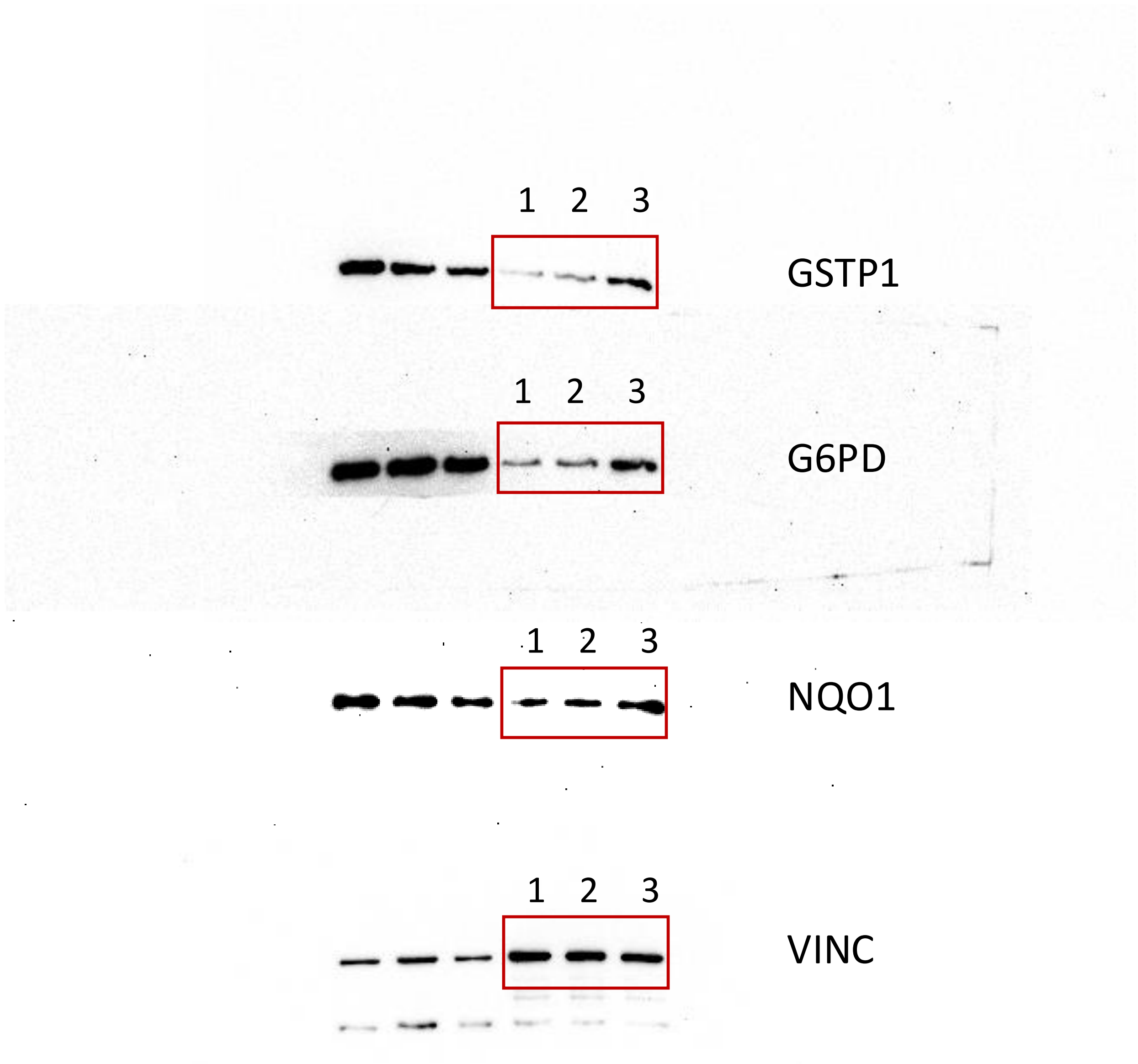

1: RNT

2: RNT + Veh

3: RNT + LN 100uM 24h

Figure 2B (bis)

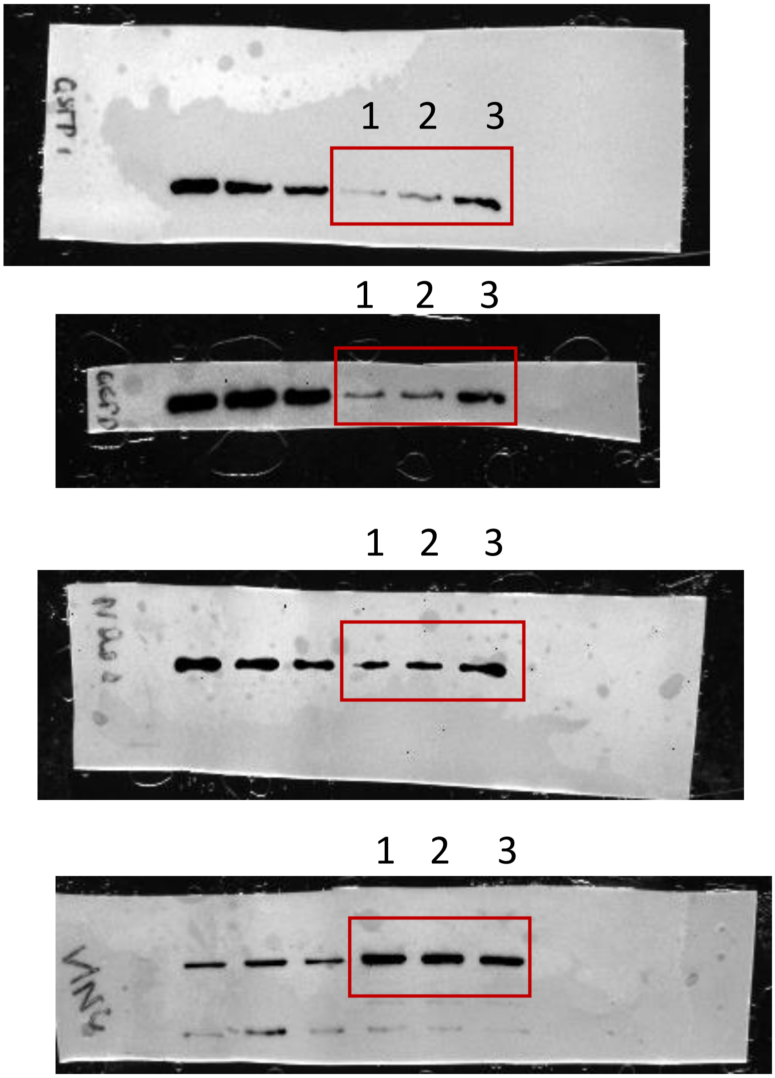

1: RNT

2: RNT + Veh

3: RNT + LN 100uM 24h

Figure 5B

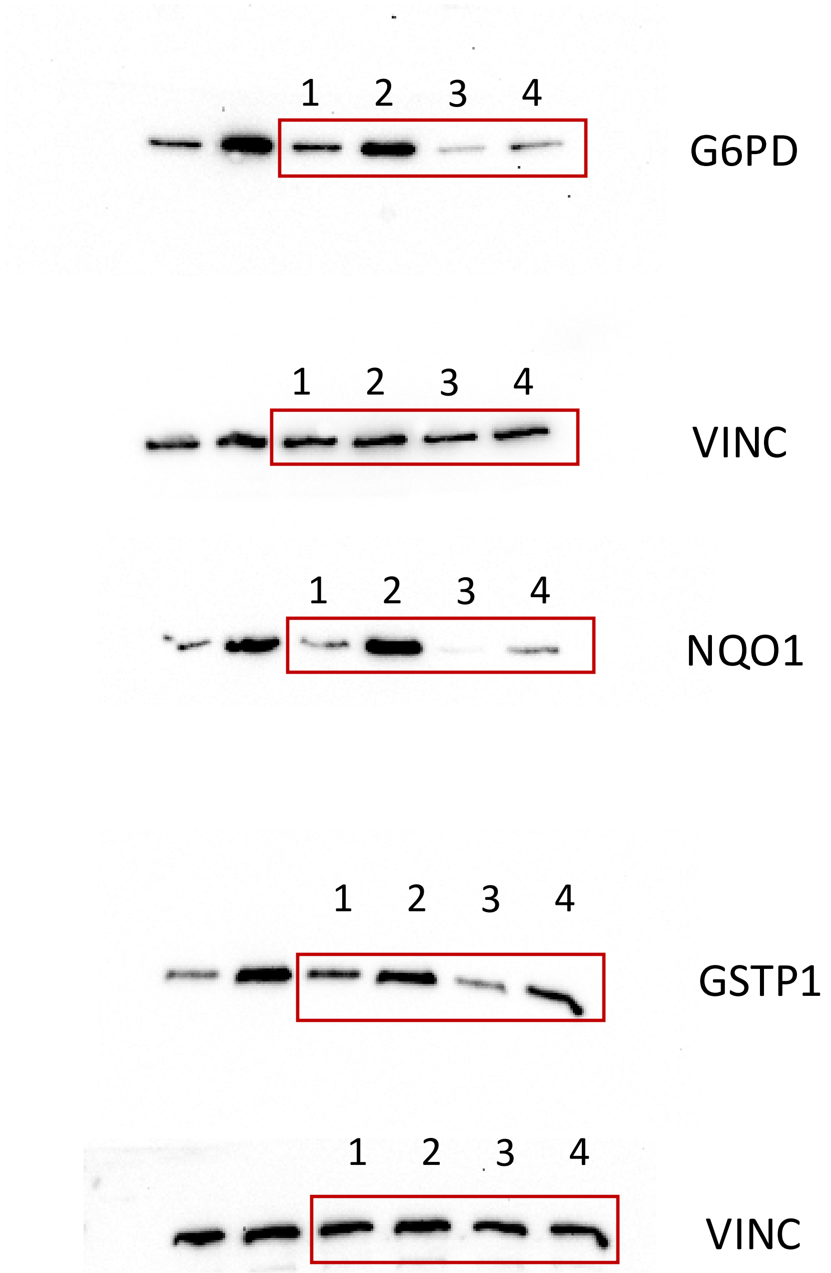

1: RNT siCTRL

2: RNT siCTRL + LN 100uM 24h

3: RNT siNRF2

4: RNT siNRF + LN 100uM 24h

Figure 5B (bis)

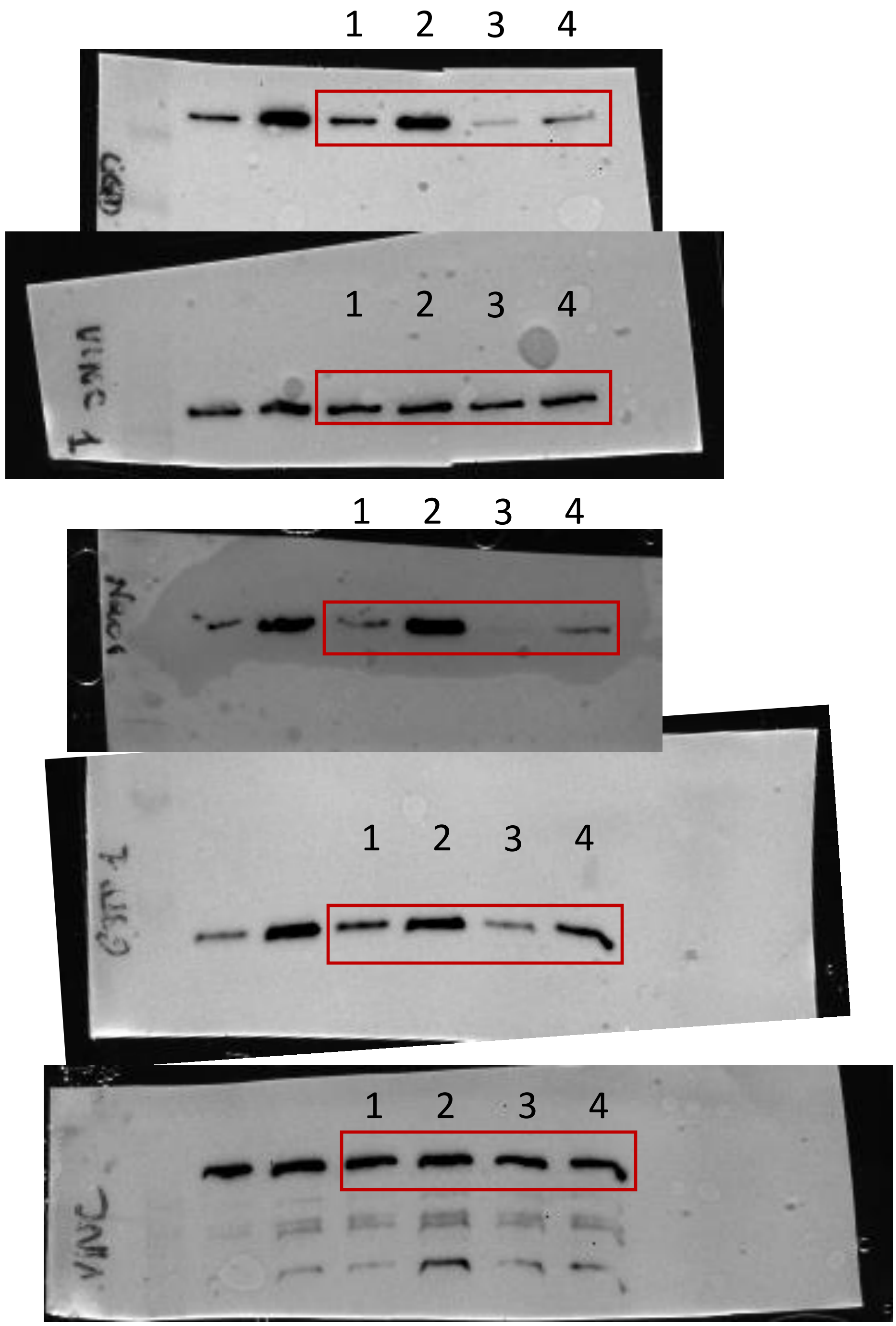

1: RNT siCTRL

2: RNT siCTRL + LN 100uM 24h

3: RNT siNRF2

4: RNT siNRF + LN 100uM 24h

Figure S1

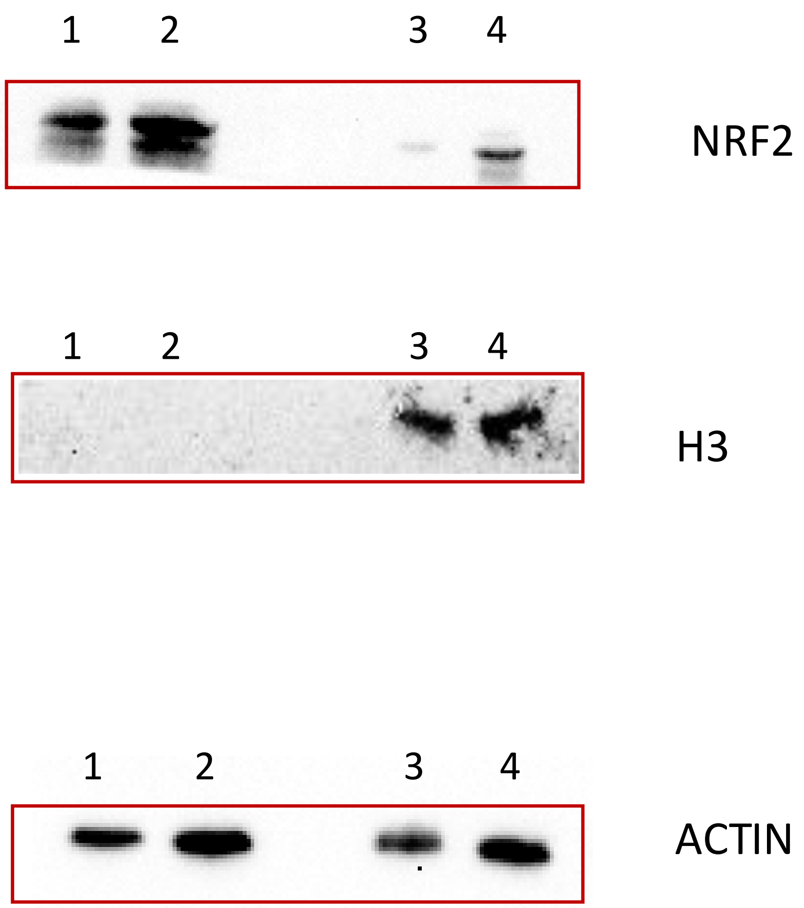

1: RNT cytoplasmic fraction NT

2: RNT cytoplasmic fraction + LN

3: RNT nuclear fraction NT

4: RNT nuclear fration + LN

Figure S1 (bis)

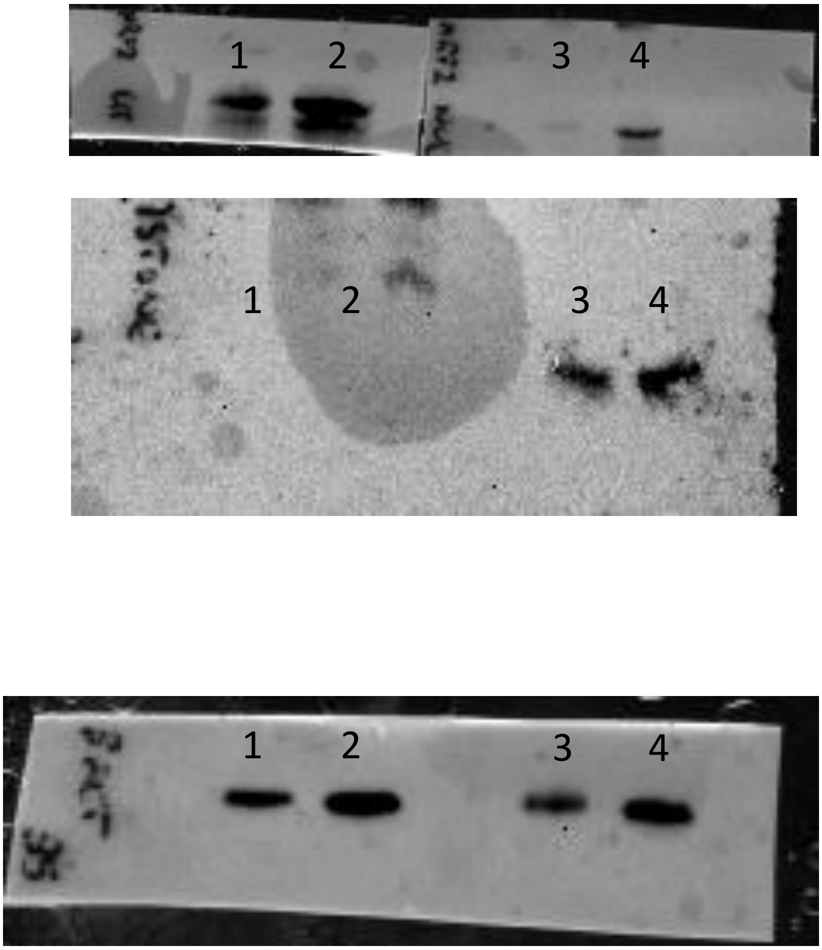

1: RNT cytoplasmic fraction NT

2: RNT cytoplasmic fraction + LN

3: RNT nuclear fraction NT

4: RNT nuclear fration + LN

Figure 2I

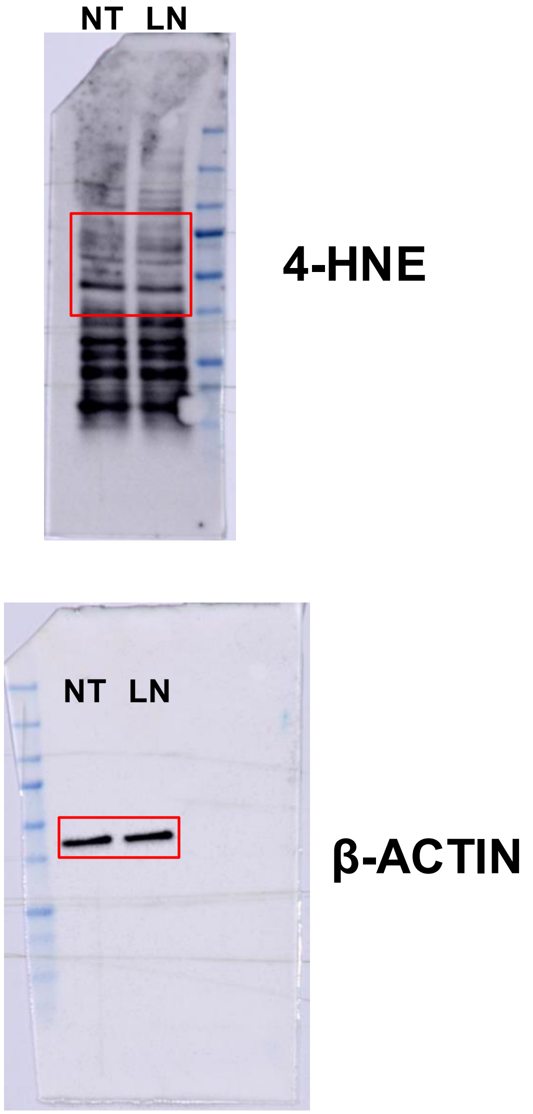

Figure 6B

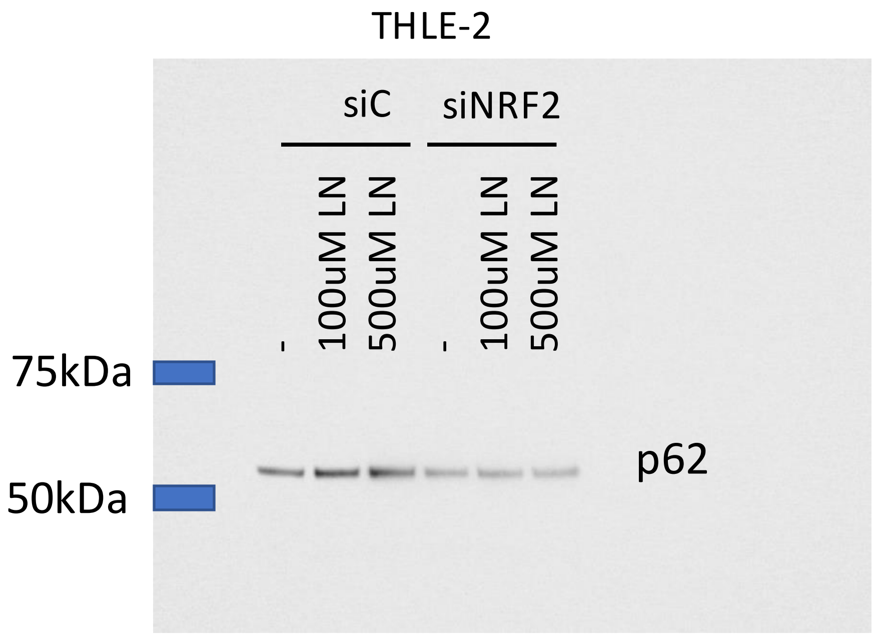
THLE-2
siC
siNRF2
-
100uM LN
500uM LN
-
100uM LN
500uM LN
75kDa
50kDa
p62

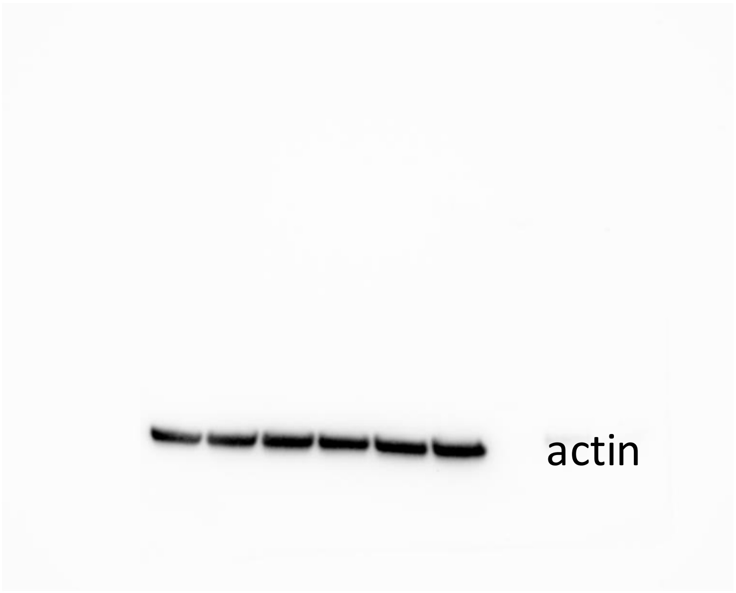
actin
